# Application of ESMACS binding free energy protocols to diverse datasets: Bromodomain-containing protein 4

**DOI:** 10.1038/s41598-019-41758-1

**Published:** 2019-04-12

**Authors:** David W. Wright, Shunzhou Wan, Christophe Meyer, Herman van Vlijmen, Gary Tresadern, Peter V. Coveney

**Affiliations:** 1 Centre for Computational Science, Department of Chemistry, University College London, London, WC1H 0AJ United Kingdom; 2 Janssen Research & Development, Turnhoutseweg 30, B-2340 Beerse, Belgium

## Abstract

As the application of computational methods in drug discovery pipelines becomes more widespread it is increasingly important to understand how reproducible their results are and how sensitive they are to choices made in simulation setup and analysis. Here we use ensemble simulation protocols, termed ESMACS (enhanced sampling of molecular dynamics with approximation of continuum solvent), to investigate the sensitivity of the popular molecular mechanics Poisson-Boltzmann surface area (MMPBSA) methodology. Using the bromodomain-containing protein 4 (BRD4) system bound to a diverse set of ligands as our target, we show that robust rankings can be produced only through combining ensemble sampling with multiple trajectories and enhanced solvation via an explicit ligand hydration shell.

## Introduction

The discovery and design of novel drugs is immensely expensive, with one study putting the cost of each new therapeutic molecule that reaches the clinic at US$1.8 billion^1^. A diversity of computational approaches, specifically binding free energy calculations which rely on physics-based molecular dynamics simulations (MD) have been developed^2^, and blind tests show that many have considerable predictive potential^3,4^. In this context, recent developments in algorithms and hardware that have reduced the cost and time of these computational approaches have seen an increase in their appeal to the pharmaceutical industry^5–9^. With commercial approaches that claim accuracy of below 1 kcal mol^−1^ now on the market^10^ it is becoming of increasing interest to understand the accuracy of and uncertainties inherent in different approaches^11^. These concerns echo wider interest in the scientific community in the lack of reproducible results in the published literature^12,13^.

One of the most common computational binding affinity prediction techniques is molecular mechanics Poisson–Boltzmann surface area (MMPBSA)^14^. This is an approximate post-processing end-state method, which uses continuum solvent models to reduce the computational cost of obtaining results. The speed and ease of setup (compared to rigorous free energy calculations) make MMPBSA an attractive candidate for use throughout the drug discovery pipeline. However, results are often seen to be system dependent and are widely perceived to be less accurate than those obtained from more expensive and theoretically rigorous approaches (such as free energy perturbation, FEP, and thermodynamic integration, TI)^2,15^. Furthermore, the term MMPBSA as used in the literature permits a wide range of variants which incorporate different sampling strategies (for example, all ligand conformers can be drawn from simulation of the complex or from independent runs) and differing solvation and entropy terms. Our previous work has demonstrated that MMPBSA analysis of single simulations is highly unreliable with calculations initiated from the same structures varying by up to 12 kcal mol^−1^ for small molecules bound to HIV-1 protease and even more for flexible ligands binding to MHC^16,17^. This served as the inspiration for our ESMACS (enhanced sampling of molecular dynamics with approximation of continuum solvent) protocols which use ensemble simulations that have been shown to produce results with reproducible uncertainties of less than 2 kcal mol^−1^ for a range of systems^9,16,18^. In this work we seek to assess the performance of the approach in a challenging dataset containing a highly varied set of ligands which interact with water in the protein binding site. We assess the impact on protocol performance of multiple trajectory sampling, ligand parameterization, inclusion of explicit water molecules and a recently developed approach to calculating the entropic contribution to the binding free energy.

The target of our investigation is the bromodomain-containing protein 4 (BRD4). Bromodomains are a major and rapidly evolving focus for the pharmaceutical industry with inhibitors targeting them having shown promising pre-clinical efficacy in pathologies ranging from cancer to inflammation. BRD4, in particular, has recently become something of a benchmark system for free energy calculations^15,19–21^, including for those based on MMPBSA^22^.

## Computational Methods

The principle behind the ESMACS family of protocols is that many short simulations provide better sampling than single long simulations, facilitating the rapid and reproducible calculation of binding affinities using variations of MMPBSA. The ESMACS simulation and analysis workflow has been automated using the Binding Affinity Calculator (BAC)^23^ which we have recently enhanced using Radical Cybertools^24,25^ to create HTBAC^26^. The goal of HTBAC is to provide a programmable interface to create computational pipelines built from selected software tools and services, and execute them on remote resources. It automates much of the complexity of running and marshalling the molecular dynamics simulations, as well as collecting and analyzing data.

Our ESMACS protocols are flexible, allowing for the analysis to be tailored to the target system. Previous targets we have studied include small molecule inhibitors of HIV proteins^18,27,28^, kinases^8,29^ and larger more flexible ligands such as peptides which bind to MHC^17^. In all these studies correlation coefficients of better than 0.7 were obtained. MMPBSA is most commonly used to assess binding affinities from a single trajectory of a protein bound to its target ligand but in this work we explore the influence of protein and ligand flexibility using independent trajectories.

### Free energy of binding computations

When two reactants combine at constant temperature and pressure the binding affinity is characterized by the change in Gibbs free energy, Δ*G*. MMPBSA is an endpoint free energy calculation; in such methods Δ*G* is calculated using:

1 $${\rm{\Delta }}G=\langle {G}_{complex}\rangle -\langle {G}_{receptor}\rangle -\langle {G}_{ligand}\rangle ,$$

where $\langle {G}_{complex}\rangle$, $\langle {G}_{receptor}\rangle$ and $\langle {G}_{ligand}\rangle$ are the average values of the Gibbs free energy for the complex, receptor (protein) and ligand respectively.

Sampling of the complex and its two components can be performed independently or conformations of the receptor and ligand extracted from simulation of the complex. The latter approach is more commonly used due to its improved convergence behaviour, a consequence of cancellation between the noisy terms describing the internal energy of the ligand, receptor and complex^30^. However, recent work has indicated that adaptation energies associated with confining the receptor and ligand in a complex can differ significantly even for closely related complexes^9^. Here we investigate a range of ESMACS protocols incorporating different component sampling strategies. When both the receptor and ligand contributions are computed from the complex trajectory we designate this a “1traj protocol”. When all three derive from independent trajectories we refer to this as the “3traj protocol” and when only one or other of the receptor or ligand contributions do so a “2traj protocol”. A suffix (either -fl or -fr, for flexible ligand and receptor respectively) is added to the protocol name to signify which component is derived from the independent simulation. Additional variants involve the use of the average receptor contribution across the complex simulations for all comparable ligands, which is indicated with an –ar (averaged receptor) suffix in the protocol name. A summary of all of the protocols, describing from which simulation component data is obtained, is given in Table 1. It should be noticed that the statistical performance of the pair of protocols 1traj-ar and 2traj-fr, and 2traj-ar and 3traj are the same, as the receptor contribution in all cases is constant. Consequently, we do not analyze the 3traj or 2traj-fr protocols explicitly.

**Table 1:** Summary of the origin of component contributions in 6 ESMACS protocols indicating whether they come from the ensemble of simulations run for the complex (C) or separate ensembles performed for the receptor (R) and ligands (L).

Protocol	Contribution to the binding free energy		
	Complex	Receptor	Ligand
1-traj	C	C	C
1-traj-ar	C	Constant	C
2-traj-fr	C	R	C
2-traj-fl	C	C	L
2-traj-ar	C	Constant	L
3-traj	C	R	L

Constant refers to the use of a constant, usually the average value across the studied systems.

The binding free energy change calculated by MMPBSA (${\rm{\Delta }}{G}_{MMPBSA}$) can be broken down into a number of components:

2 $${\rm{\Delta }}{G}_{MMPBSA}={\rm{\Delta }}{G}_{ele}^{MM}+{\rm{\Delta }}{G}_{vdW}^{MM}+{\rm{\Delta }}{G}_{{\rm{i}}{\rm{n}}{\rm{t}}}^{MM}+{\rm{\Delta }}{G}_{nonpol}^{sol}+{\rm{\Delta }}{G}_{pol}^{sol},$$

where ${\rm{\Delta }}{G}_{ele}^{MM}$, ${\rm{\Delta }}{G}_{vdW}^{MM}$ and ${\rm{\Delta }}{G}_{{int}}^{MM}$ are the electrostatic, van der Waals and the internal bonded contributions to the molecular mechanics free energy difference, respectively, and ${\rm{\Delta }}{G}_{pol}^{sol}$ and ${\rm{\Delta }}{G}_{nonpol}^{sol}$ are the polar and non-polar solvation terms, respectively.

The MMPBSA.py^31^ program, provided as part of the AmberTools 14 package^32^, was used in the evaluation of all components of the MMPBSA calculation. The electrostatic free energy of solvation, ${\rm{\Delta }}{G}_{pol}^{sol}$, is the part of the calculation described by the Poisson-Boltzmann (PB) calculation. Default values were used for the PB calculation (grid spacing of 0.5 Å, internal and external dielectric constants of 1 and 80, respectively). The non-polar solvation free energy calculation is calculated from the solvent accessible surface area using the traditional one component method (specified using inp = 1 in the input file). In this approach the surface tension, *γ*, is set to 0.00542 kcal mol^−1^ Å^−2^) and the off-set, *β,* to 0.92 kcal mol^−1^. The fill ratio parameter was set to 4.0 which does not impact the results but ensures the stability of the calculations. For calculations in which explicit water molecules were incorporated as part of the receptor, the closest *N* molecules to the ligand were chosen for inclusion.

#### Entropic contribution to binding free energies

A variety of options are available to incorporate entropic contributions to Δ*G*. The most common approach is normal mode analysis^33,34^ but it can require similar computational effort to the underlying simulations in order to obtain converged results^18^. Consequently, here we explore the use of another, more computationally efficient, alternative approach proposed by Duan *et al*.^35^. In their formulation the “variational entropy” can be derived from the fluctuations of the receptor-ligand interaction energy, *E*^*inter*^. This energy can be calculated using components of the MMPBSA calculation:

3 $${E}^{{inter}}={G}_{ele}^{MM}+{G}_{vdW}^{MM}.$$

The fluctuation in interaction energy is then given by:

4 $${\rm{\Delta }}{E}^{{inter}}={E}^{{inter}}-\langle {E}^{{inter}}\rangle ,$$

where angle braces indicate an ensemble average. This is then used to compute the entropic contribution to binding via:

5 $$-T{\rm{\Delta }}{S}_{{var}}={k}_{B}T\,\mathrm{ln}\,\langle {e}^{\beta {\rm{\Delta }}{E}^{{inter}}}\rangle $$

where *k*_*B*_ is the Boltzmann constant and $\beta =1/{k}_{B}T$.

### Simulation setup

Ensembles of 25 replica MD simulations were conducted using the package NAMD 2.11^36^ for each system (complex, receptor or ligand) studied. All simulations were conducted using the protocol incorporated into BAC^23^. We have previously shown that the use of 25 replica ensembles provides a good balance of computational cost and calculation uncertainty for a number of varied systems^8,17,18^.

**Figure 1:**
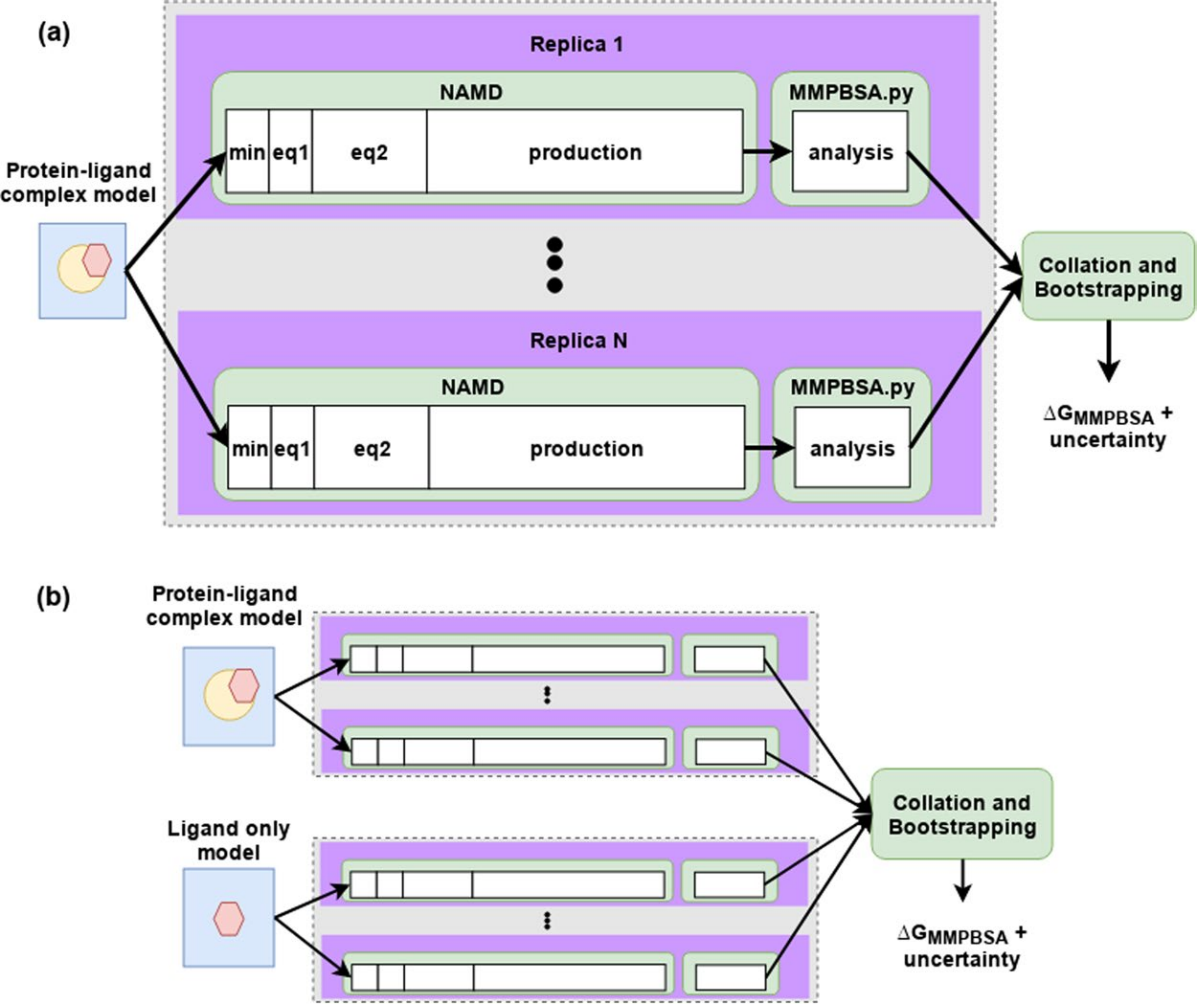
Overview of the ESMACS workflow. The 1traj protocol is shown in (**a**) consisting of an ensemble of 1 to N (25 in this study) simulations of the protein-ligand complex. Each simulation is made up of (min)imization and two (eq)uilibration steps and a single production NAMD run which are each analyzed independently using the MMPBSA.py script. The output of the analysis is then collated and bootstrap statistics produced. The multiple trajectory approaches, shown in (**b**) follow a similar outline but with independent trajectories also run of the ligand system alone.

Each system was minimized with all heavy protein atoms restrained at their initial positions (with a restraining force constant of 4 kcal mol^−1^ Å^−2^). Initial velocities were then generated independently for each replica from a Maxwell–Boltzmann distribution at 50 K. Each system was virtually heated to 300 K over 60 ps and subsequently maintained at this temperature using a thermostat (employing a coupling coefficient of 1 ps^−1^) during which time the restraints applied during minimization were retained. Once the system reached the correct temperature the pressure was maintained at 1 bar using a Berendesen barostat (with a pressure coupling constant of 0.1 ps). Subsequent to the heating, a series of equilibration runs, totaling 2 ns, were conducted, during which the restraints on heavy atoms were gradually reduced. The restraint reduction occurs in ten 100 ps steps, after each one the force constant was halved. Finally, 4 ns production simulations were executed with snapshots output for analysis every 100 ps. A 2 fs time step was used for all MD simulation steps. The workflow of the ESMACS protocols is shown in Fig. 1. For each system run through the 1traj protocol an ensemble of independent NAMD simulations is executed, consisting of four steps. The first minimization (min), which is followed by two equilibration steps (labelled eq1 and eq2 respectively). In Eq. 1 the system is heated while restraints are applied to heavy atoms. In Eq. 2 restraints are gradually reduced before free simulation is undertaken. After 2 ns of aggregate equilibration the 4 ns production phase is initiated. It is the production trajectory which is analysed by MMPBSA.py. A script is then run to aggregate these results from the ensemble of simulations and values of Δ*G*_*MMPBSA*_ computed along with bootstrap statistics. In multiple trajectory approaches a second ensemble of ligand-only simulations is conducted and fed into the aggregation and bootstrapping script. Full simulation details are provided in the main text.

### Experimental Datasets

**Figure 2:**
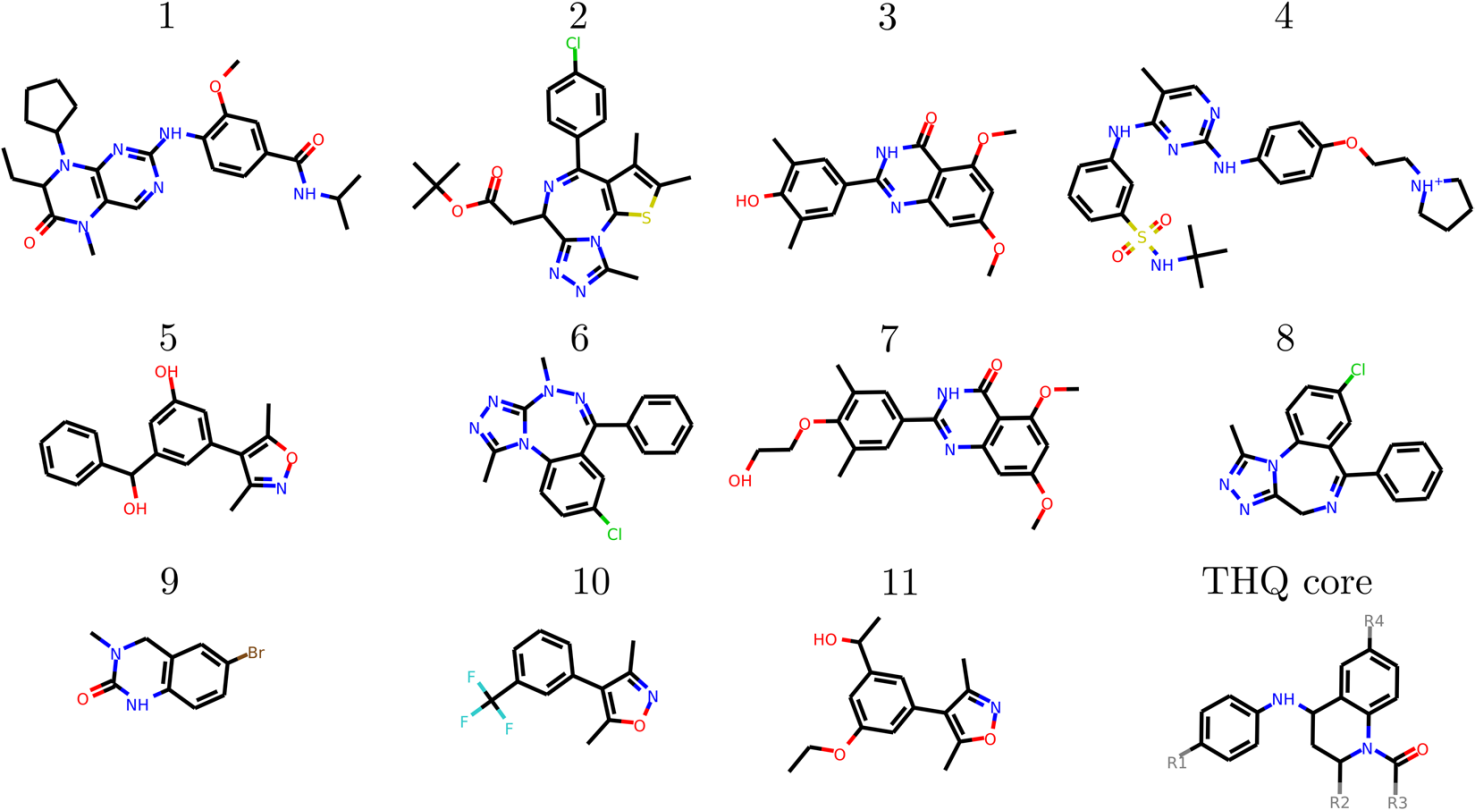
Chemical structures of ligands from the BRD4 dataset previously studied by Aldeghi *et al*.^15^ (1–11) and the tetrahydroquinoline (THQ) scaffold. Full R-group information for the THQ dataset ligands is provided in Fig. 3 and Table 2.

**Figure 3:**
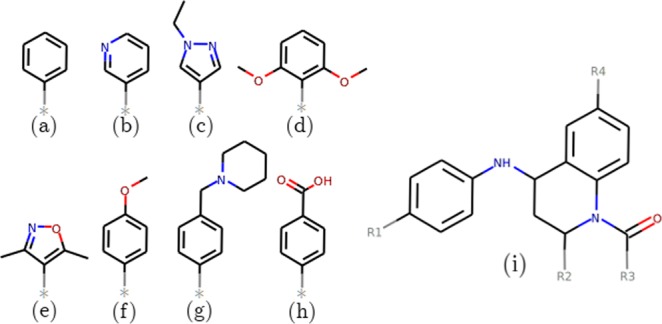
(**a**–**h**) Structures of the groups of the side groups which are added to the tetrahydroquinoline (THQ) scaffold shown in (**i**) to create the ligands in the THQ dataset. Full composition information for the ligands is provided in Table 2.

This study investigates a combination of BRD4 ligand binding datasets which have been the subjects of earlier studies. The first, previously studied by Aldeghi *et al*.^15^ using a combination of FEP based absolute binding free energy and MMPBSA techniques, contains a diverse set of 11 ligands which will be referred to as the diverse (DIV) dataset. The second was recently studied by our group in collaboration with GlaxoSmithKline^9^ (using a combination of ESMACS and ensemble thermodynamic integration approaches) and contains 16 ligands, all based on a single tetrahydroquinoline (THQ) template (consequently we identify this as the THQ dataset). The compounds were selected to represent a range of chemical functionality and binding affinities, despite their shared scaffold. The first 11 compounds are labeled 1 to 9 according to the scheme used by Aldeghi *et al*.^15^, the THQ based ligands are labeled THQ1 to THQ16 (the numbers correspond to those used in Wan *et al*.^9^). The chemical structure of the first 11 compounds and the THQ scaffold are shown in Fig. 2. Details of the groups found at positions R1 to R4 in the THQ based ligands are detailed in Fig. 3 and Table 2. Ligand 4 was parameterized with a charge of +1. Compounds THQ10 to THQ12 and THQ16 are positively charged (+1), and compounds THQ13 to THQ15 are negatively charged (−1).

**Table 2:** Composition of the ligands of the THQ dataset.

Ligand ID	R1	R2	R3	R4
THQ1	H	Me	Me	(a)
THQ2	H	Me	Me	H
THQ3	H	Me	Me	(f)
THQ4	H	Me	Me	(b)
THQ5	H	Me	Me	(c)
THQ6	H	Me	Me	(d)
THQ7	H	Me	Me	(e)
THQ8	H	Me	Et	(f)
THQ9	H	Me	i-Pr	(f)
THQ10	H	Me	Me	(g)
THQ11	H	Et	Me	(g)
THQ12	H	Pr	Me	(g)
THQ13	H	Pr	Me	(h)
THQ14	H	Et	Me	(h)
THQ15	Cl	Me	Me	(h)
THQ16	H	Me	Me	(g)

The groups found at R4 are shown in Fig. 3(a–h), with the common THQ scaffold in Fig. 3(i). Compounds THQ1–9 are neutral, 10–12 and 16 are positively charged (+1), and 13–15 negatively charged (−1). All compounds are the 2-(S) 4-(R) isomers except compound 16 which is 2-(R) 4-(S).

Experimental binding free energies (Δ*G*_*expt*_) for the first dataset were obtained from a combination of SPR, Alphascreen and Isothermal Titration Calorimetry (ITC) experiments^15^, whereas those for the THQ dataset are derived from IC_50_ values from FRET^9^. These techniques are very different from one another and will necessarily introduce varying levels of uncertainty into the data they provide. The divergence in the origin of the measurements is representative of the sources of experimental data to which free energy calculations are typically compared. This, alongside the lack of rigorously derived uncertainty estimates in the experimental data, must be borne in mind when assessing protocol performance. In Table 3

**Table 3:** Experimental binding affinities for both the diverse (DIV) and tetrahydroquinoline scaffold (THQ) datasets.

Ligand ID	pIC_50_	Δ*G*_*expt*_ (kcal mol^−1^)
**Diverse (DIV)**		
1	—	−9.8 (0.1)
2	—	−9.6 (0.1)
3	—	−9.0 (0.1)
4	—	−8.9 (0.1)
5	—	−8.8 (0.1)
6	—	−8.2 (0.1)
7	—	−7.8 (0.1)
8	—	−7.4 (0.1)
9	—	−7.3 (0.1)
10	—	−6.3 (0.1)
11	—	−5.6 (0.1)
**Tetrahydroquinoline scaffold (THQ)**		
THQ1	7.0	−9.6 (0.1)
THQ2	5.6	−7.7 (0.1)
THQ3	6.8	−9.3 (0.1)
THQ4	6.8	−9.3 (0.1)
THQ5	7.9	−10.8 (0.1)
THQ6	5.6	−7.7 (0.1)
THQ7	5.8	−8.0 (0.1)
THQ8	6.5	−8.9 (0.1)
THQ9	<4.3	>−5.9 (0.1)
THQ10	7.6	−10.4 (0.4)
THQ11	6.8	−9.3 (0.1)
THQ12	5.5	−7.5 (0.1)
THQ13	5.4	−7.4 (0.1)
THQ14	6.7	−9.2 (0.3)
THQ15	7.8	−10.7 (0.1)
THQ16	5.4	−7.4 (0.4)

The values used here were taken from Aldeghi *et al*.^15^ for the DIV and Wan *et al*.^9^ for the THQ datasets respectively. Values for ligands 1–4 and 6–8 were derived from ITC experiments, 5 from SPR and 9–11 from Alphascreen. All THQ values were derived from IC_50_ values from FRET experiments.

we provide the full experimental binding affinities for both the diverse (DIV) and tetrahydroquinoline scaffold (THQ) datasets.

### Structural models

**Figure 4:**
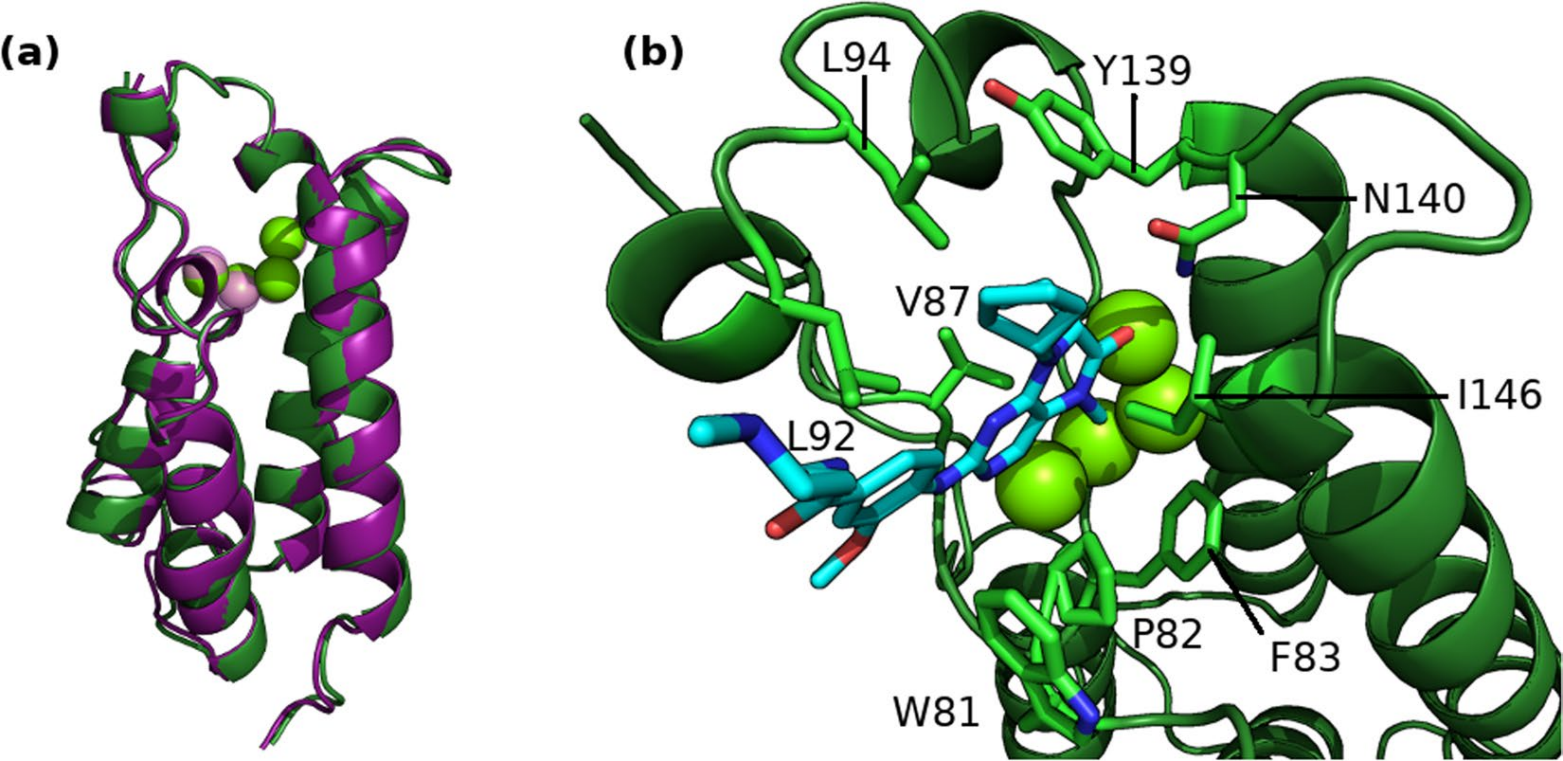
BRD4 structure in cartoon representation with conserved binding site waters shown as van der Waals spheres. (**a**) A comparison of the structural models created as derived from the 2OSS (green) and 4BJX (purple) PDBs. (**b**) The initial binding mode of ligand 1 (shown in chemical representation) in the 2OSS derived BRD4 structure.

The ligands from both datasets were simulated bound to the two BRD4 structural models based on PDBs 2OSS and 4BJX respectively (these are the initial structures used in Aldeghi *et al*.^15^ and Wan *et al*.^9^). The former represents the apo BRD4 and the latter the protein bound to a THQ based ligand. The secondary structure of both models is very similar (see Fig. 4a) and the RMSD between the two structures is 0.44 Å. All crystallographic water molecules were retained, including four which are conserved in the binding site of both models. The poses of the ligands in the DIV dataset were extracted from crystal structures (PDBs: 3U5J, 3U5L, 4OGI, 4OGJ, 3MXF, 4MR3, 4MR4, 3SVG, 4J0R and 4HBV), except for one ligand, labelled 10, which was modeled (based on PDB 3SVG) and docked into 2OSS as two conformers. These are the same two conformers used in Aldeghi *et al*.^15^, differing by a 180° flip of the trifluorophenyl moiety. The modelled poses were aligned and copied into the 4BJX based models. Poses of the THQ ligands were based on that of I-BET726 as found in the 4BJX structure.

System setup, including the creation of a water box and addition of neutralizing ions, was performed using AmberTools 17^37,38^. The majority of simulations were conducted using protein parameters taken from the standard Amber force field for bioorganic systems (ff14SB)^39^. Reproducibility studies of the THQ ligands were conducted using an earlier version of the forcefield, ff99SBildn^40^.

Drug parameters were produced using the general Amber force field (GAFF)^41^. The majority of the simulations presented here employ ligands prepared using the Gaussian/RESP protocol. In this approach, Gaussian 98^42^ was used to perform geometric optimization of the inhibitor with 6–31G** basis functions, and the restrained electrostatic potential (RESP) procedure was used to calculate the partial atomic charges. Reproducibility studies of the DIV dataset were conducted using AM1-BCC^43^ derived charges. All charge assignment and input file generation was performed in the Antechamber component of AmberTools.

### Statistics and uncertainties

All statistics presented use their standard definitions with the exception of the mean unsigned error (MUE). It is well known that MMPBSA results have a significant offset from experimental values (typically of the order of 15 to 25 kcal mol^−1^) due to a range of factors, in particularly the neglect of entropic contributions^33,34^. Consequently we present values corrected for the systematic (mean signed) error and designate them cMUE.

We compute uncertainties for all metrics through bootstrapping analysis. This method involves resampling with replacement the N input data points (in this case, the replica averages of Δ*G*_*MMPBSA*_) to provide a new bootstrap sample also containing N data points. This process is repeated many times (in our case 5000 times) and the statistic of interest of each bootstrap population calculated. The standard deviation of these values provides an estimate of the uncertainty associated with an average derived from a given sample; this is what is quoted as the bootstrap error measure of our statistics. For correlation coefficients samples are drawn from the overall averages for each ligand paired with the relevant experimental value. In addition to this metric, when making a direct comparison of specific correlation coefficients we will also quote 95% confidence intervals. These intervals are calculated by sorting the bootstrap sample distribution of correlation coefficients and taking the values falling at the 2.5 and 97.5 percentiles.

## Results

Here we evaluate the performance of a range of ESMACS protocols in reproducing the experimental rankings across the full diverse ligand dataset, the robustness of this ranking to choices in system setup and the influence of non-standard MMPBSA components.

### Standard ESMACS Performance and Robustness to Initial Structure Variation

Comparison of the results of all ESMACS protocols across the full DIV + THQ dataset shows a distinct trend in which inclusion of the receptor average energy considerably improves the predictions obtained for both initial protein models. In both cases 1traj results have a Spearman rank coefficient, *r*_*s*_, of 0.46 [CI: 0.16–0.84 for both] which improves to 0.66 [CI: 0.50–0.94]/0.60 [CI: 0.40–0.91] (2OSS/4BJX) when both ligand and receptor flexibility are accounted for in the 2traj-ar protocol. In the DIV dataset better ranking can be obtained using receptor flexibility alone, but in order to obtain good rankings for THQ both additional contributions are required. This is the same behaviour observed in the simulation results for the THQ dataset in Wan *et al*.^9^; however the overall ranking is worse (the original study obtained an *r*_*s*_ of 0.78 [CI: 0.53–0.92]), primarily due to the stronger predicted binding affinity for the experimentally least potent drug, THQ16, in the present study.

**Figure 5:**
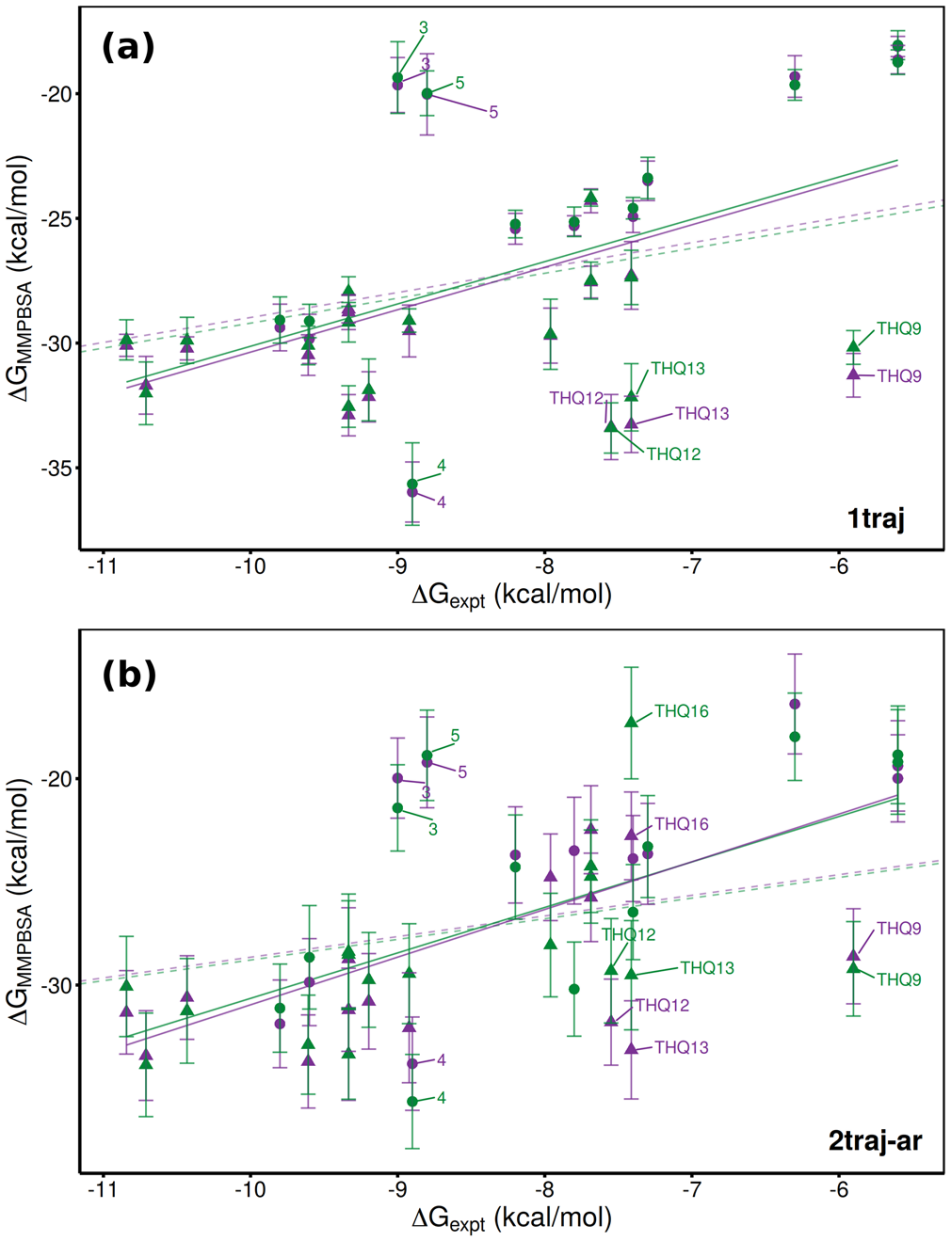
Comparison of experimental and computed binding affinities for the combined DIV (circle) and THQ (triangle) datasets. Computational results were obtained using (**a**) 1traj and (**b**) 2traj-ar MMPBSA based ESMACS protocols. Results are shown for simulations initiated from models based on PDBs 2OSS (green) and 4BJX (purple). Solid lines represent lines of best fit, dashed ones optimal correlations.

The improvement between 1traj and 2traj-ar is illustrated in Fig. 5, which shows that outliers are moved closer to the overall trend line (particularly apparent for the DIV ligands 3, 4 and 5 which were also outliers in Aldeghi *et al*.^15^). These three ligands have similar experimental binding energies but a difference of 15 kcal mol^−1^ in 1traj and 10 kcal mol^−1^ in 2traj-ar is seen in Δ*G*_*MMPBSA*_ The ranking improvement is larger for the THQ ligands than the DIV dataset, with the 1traj results exhibiting little if any correlation with experiment. The main THQ outliers in the 1traj results are THQ12, THQ13 and THQ9. The first two are moved closer to the trend in the 2traj-ar results but THQ9 remains more negative than might be expected. Another feature of the 2traj-ar data here is that greater separation is seen between the results obtained from the two BRD4 structures for TH12, THQ13 and most pronouncedly THQ16. This is in contrast to nearly all other ligands where the values obtained from simulations with either model are well within the error margin, many sitting on top of one another in Fig. 5.

It can also be seen in Table 4 that the impact of the incorporation of receptor ‘strain’ in the 1traj-ar and 2traj-ar protocols is different in the DIV and THQ subsets. In the 4BJX simulations the DIV rankings are notably less good than that in the 1traj, whilst they are fairly similar in the 2OSS case. Whereas for THQ, we find that accounting for the receptor and ligand flexibility is necessary to obtain a good ranking in both cases. Overall the results from the 2OSS structure are better than those from 4BJX. However, it should be noted that the Δ*G*_*MMPBSA*_ values for all drugs using the 1traj protocol agree within error (see Fig. 5a).

**Table 4:** Performance of different MMPBSA based ESMACS protocols in reproducing experimental binding free energies, measured by mean unsigned error (MUE), Pearson’s predictivity index (PI), correlation coefficient (*r*) and Spearman’s rank coefficient (*r*_*s*_).

Protocol	Dataset	cMUE*		PI	r		r_s_	
**2OSS Structure**								
1traj	DIV + THQ	3.25	(0.51)	0.48	0.51	(0.16)	0.46	(0.18)
	DIV	3.38	(0.68)	0.74	0.64	(0.19)	0.69	(0.23)
	THQ	2.01	(0.37)	0.11	0.19	(0.22)	0.09	(0.27)
1traj-ar	DIV + THQ	3.30	(0.49)	0.62	0.61	(0.14)	0.60	(0.14)
	DIV	3.98	(0.68)	0.75	0.67	(0.18)	0.72	(0.19)
	THQ	2.05	(0.30)	0.46	0.47	(0.17)	0.42	(0.22)
2traj-ar	DIV + THQ	3.57	(0.54)	0.65	0.62	(0.12)	0.66	(0.11)
	DIV	4.09	(0.78)	0.66	0.62	(0.18)	0.64	(0.21)
	THQ	2.41	(0.67)	0.67	0.54	(0.13)	0.65	(0.17)
2traj-fl	DIV + THQ	3.33	(0.59)	0.59	0.55	(0.14)	0.57	(0.14)
	DIV	3.41	(0.88)	0.75	0.59	(0.19)	0.72	(0.20)
	THQ	2.09	(0.55)	0.41	0.40	(0.17)	0.39	(0.23)
**4BJX Structure**								
1traj	DIV + THQ	3.32	(0.53)	0.48	0.50	(0.17)	0.46	(0.18)
	DIV	3.54	(0.80)	0.79	0.65	(0.18)	0.74	(0.20)
	THQ	2.14	(0.43)	0.07	0.11	(0.24)	0.04	(0.27)
1traj-ar	DIV + THQ	4.05	(0.48)	0.52	0.56	(0.14)	0.49	(0.15)
	DIV	3.51	(0.78)	0.60	0.68	(0.18)	0.55	(0.27)
	THQ	2.55	(0.46)	0.24	0.30	(0.18)	0.16	(0.25)
2traj-ar	DIV + THQ	3.97	(0.44)	0.61	0.63	(0.12)	0.60	(0.13)
	DIV	3.42	(0.82)	0.57	0.67	(0.17)	0.55	(0.24)
	THQ	2.70	(0.48)	0.51	0.51	(0.15)	0.46	(0.22)
2traj-fl	DIV + THQ	3.46	(0.51)	0.54	0.56	(0.14)	0.52	(0.16)
	DIV	3.52	(0.86)	0.75	0.63	(0.18)	0.73	(0.20)
	THQ	2.20	(0.56)	0.27	0.36	(0.19)	0.22	(0.26)

Bootstrapped error provided in brackets where appropriate. Results are provided for the diverse (DIV) and tetrahydroquinoline (THQ) datasets and both combined (DIV + THQ).

*MUE corrected for mean signed error in kcal mol^−1^.

### Robustness of Ranking to Parameterization

**Figure 6:**
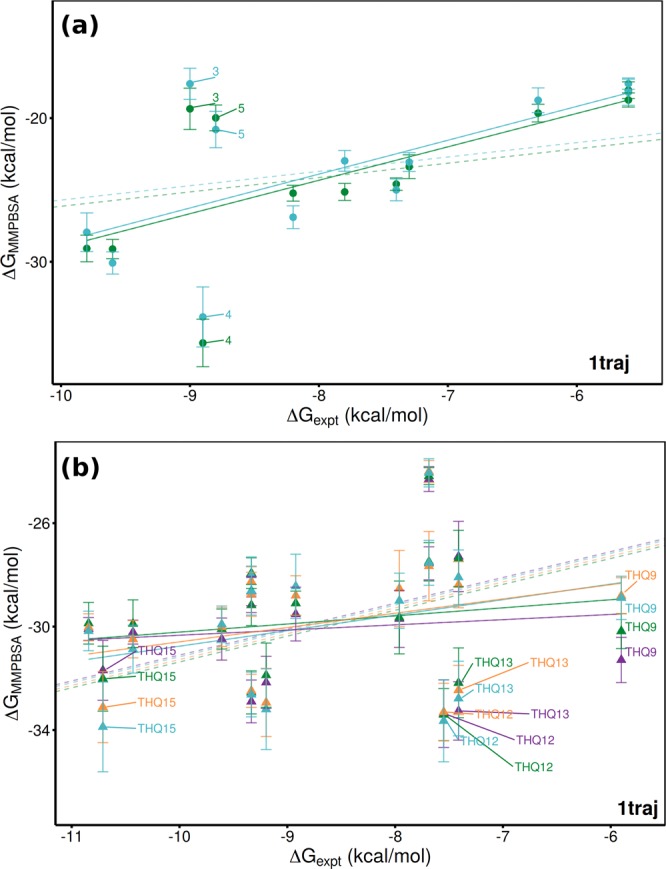
Comparison of 1traj ESMACS results using different forcefield choices. In (**a**) the ranking of the DIV dataset is shown with the same protein forcefield (ff14) but different ligand parameterization methods (Gaussian-RESP in dark green, AM1-BCC in lighter green). In (**b**) results for the THQ dataset are compared using the ff14 (dark green is based on PDB 2OSS, purple on 4BJX) and ff99ildn (cyan based on PDB 2OSS, orange on 4BJX) forcefields. Solid lines represent lines of best fit, dashed ones optimal correlations.

Two of the key decisions in ligand binding free energy calculations are the choices of the forcefield and how small molecules are parameterized. For simulations using Amber forcefields the choice of procedures for ligand preparation is usually whether to use AM1-BCC or Gaussian/RESP based protocols to determine atom charges in combination with the GAFF general purpose forcefield parameters. Following the choice in Wan *et al*.^9^ we used Gaussian/RESP for the majority of simulations in this work, but to evaluate the influence of this we re-ran the DIV dataset in the 2OSS model using the AM1-BCC methodology. Figure 6a shows that the Δ*G*_*MMPBSA*_ values for the large majority of the ligands are highly correlated between the two schemes (within 1–2 kcal mol^−1^). This and the similar correlation with experiment (shown in Table 5) indicates that our results are robust with respect to this choice.

**Table 5:** Performance of different MMPBSA based ESMACS protocols in reproducing experimental binding free energies using the AM1-BCC method to parameterize ligands.

Protocol	cMUE*		PI	r		r_s_	
1traj	3.57	(0.72)	0.67	0.65	(0.20)	0.61	(0.26)
1traj-ar	5.16	(2.18)	0.62	0.42	(0.22)	0.56	(0.26)
2traj-ar	5.16	(2.22)	0.62	0.39	(0.21)	0.56	(0.27)
2traj-fl	3.49	(0.78)	0.68	0.61	(0.19)	0.62	(0.26)

Performance is measured by mean unsigned error (MUE), Pearson’s predictivity index (PI), correlation coefficient (*r*) and Spearman’s rank coefficient (*r*_*s*_). Bootstrapped error shown in brackets where appropriate. Results are provided for the diverse (DIV) dataset alone bound to protein models based on PDB 2OSS.

*MUE corrected for mean signed error in kcal mol^−1^.

The Wan *et al*.^9^ study employed the Amber ff99ildn forcefield for the protein, whilst in this study we have used ff14. In general the results obtained for all ligands are consistent but two ligands at either end of the rankings, THQ9 and THQ15, differ significantly as shown in Fig. 6b. The ranking performance with ff99ildn is described in Table 6. Comparing to those for ff14 (the THQ subset values in Table 4) shows ff99ildn provides better results, especially those for 2traj-ar in the 4BJX model (*r*_*s*_ of 0.80 compared to 0.46). There are many factors which may cause this difference but one we identified was the possibility that the balance between direct and water mediated interactions might be altered by modifications to the amino acid side chain parameters. This in part motivated our investigation of the impact of including explicit water molecules in the receptor component of our calculations (see the following section).

**Table 6:** Performance of different MMPBSA based ESMACS protocols in reproducing experimental binding free energies using the ff99ildn protein forcefield.

Protocol	cMUE*		PI	r		r_s_	
**2OSS Structure**							
1traj	2.16	(0.39)	0.35	0.31	(0.21)	0.28	(0.25)
1traj-ar	2.62	(0.51)	0.28	0.37	(0.18)	0.25	(0.24)
2traj-ar	2.45	(0.48)	0.60	0.54	(0.13)	0.58	(0.17)
2traj-fl	2.21	(0.36)	0.53	0.50	(0.17)	0.45	(0.23)
**4BJX Structure**							
1traj	2.00	(0.38)	0.33	0.31	(0.21)	0.26	(0.24)
1traj-ar	1.73	(0.33)	0.69	0.62	(0.11)	0.58	(0.18)
2traj-ar	1.79	(0.41)	0.84	0.77	(0.07)	0.80	(0.10)
2traj-fl	2.09	(0.37)	0.55	0.51	(0.16)	0.47	(0.22)

Performance is measured by mean unsigned error (MUE), Pearson’s predictivity index (PI), correlation coefficient (*r*) and Spearman’s rank coefficient (*r*_*s*_). Bootstrapped error provided in brackets where appropriate. Results are provided for the THQ dataset alone bound to protein models based on both PDBs 2OSS and 4BJX.

*MUE corrected for mean signed error in kcal mol^−1^.

### Inclusion of Explicit Water

Aldeghi *et al*.^22^ found that the inclusion of explicit water molecules as part of the receptor in MMPBSA calculations improved the correlation with experiment in the DIV dataset. Here we explore whether this finding is reproducible using ensemble simulations and is robust to the addition of THQ ligands to the dataset under investigation. We use the same strategy in selecting water molecules for inclusion as the previous work, namely using the closest *N* to the ligand in each frame of the simulation trajectory.

**Figure 7:**
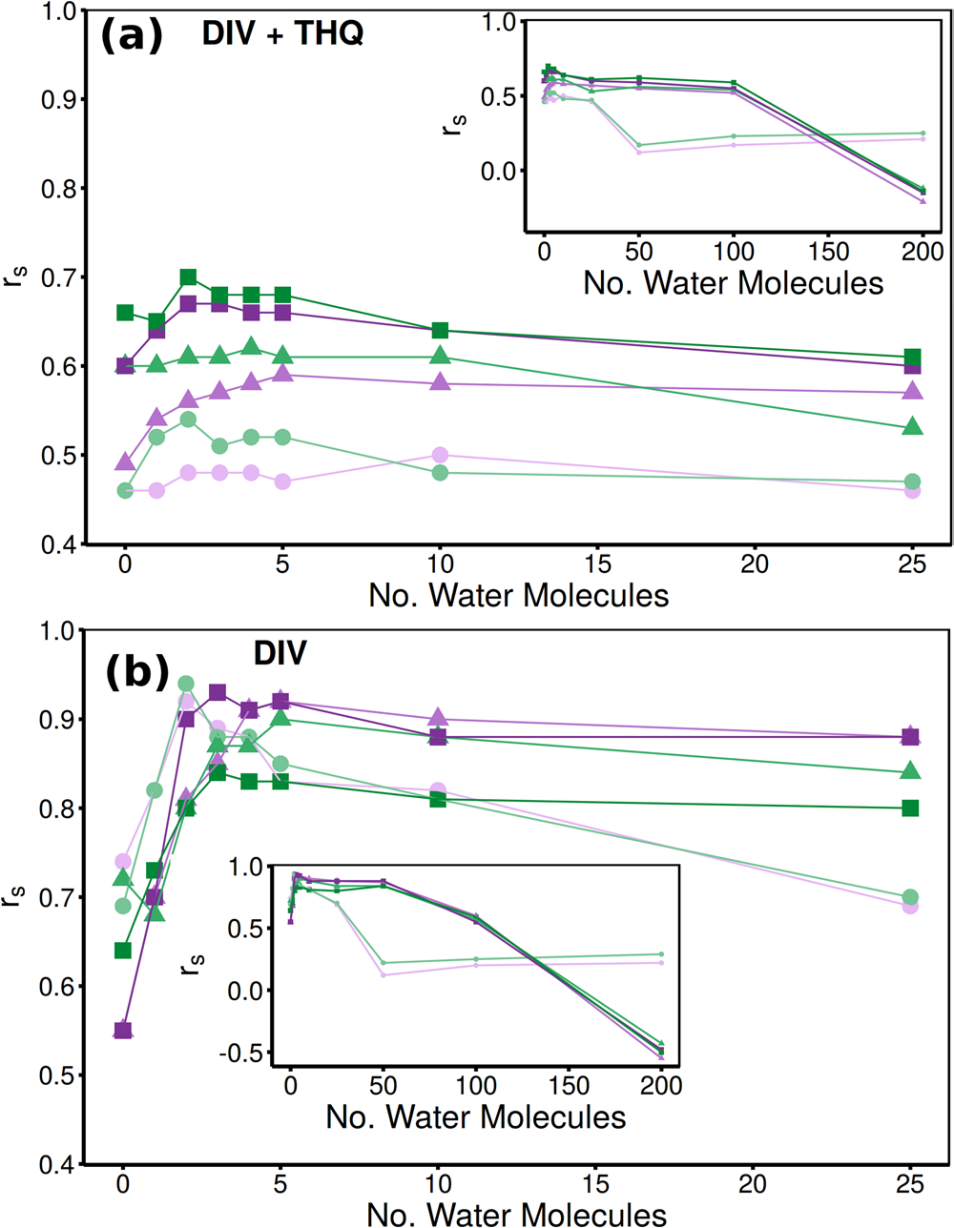
Impact of the inclusion of explicit water molecules as part of the receptor in ESMACS calculations on the Spearman rank coefficient (*r*_*s*_), exhibited for both (**a**) combined DIV and THQ and (**b**) DIV alone datasets. Results are shown for simulations initiated from models based on PDBs 2OSS (green) and 4BJX (purple) and three protocols; 1traj (circles), 1traj-ar (triangles) and 2traj-ar (squares). Main figures show detailed view of the inclusion of up to 25 water molecules, inset shows how performance falls off as 50 or more water molecules are accounted for.

We found a large difference in the impact of explicit water molecules between the combined DIV + THQ and DIV alone datasets. The correlations within the THQ dataset do not benefit from the inclusion of the additional water molecules in any protocol. For the combined dataset we find that up to around 5 explicit water molecules improves the rankings for all protocols (see Fig. 7a). After 50 water molecules are included 1traj performance drops to show no significant correlation with experiment and is only slightly improved as more molecules are added. A similar pattern is observed for the 1traj-ar and 2traj-ar results although, after the initial improvements, performance is more stable until 100 water molecules are included when an even sharper fall off is observed.

For the DIV dataset as shown in Fig. 7b the improvements are yet more marked. The biggest improvement is seen in the 1traj results. Furthermore, the MUE for these rankings does not increase with adding more water near peak performance 3.38/3.54 for 0 and 3.08/3.08 for 5 water molecules (2OSS/4BJX). In line with the results of Aldeghi *et al*.^22^ the peak performance has ${r}_{s} > 0.9$; however, unlike in the previous work here we see this at 2 water molecules included with a decline after 5 (as opposed to a peak at 20 and consistent performance thereafter). A number of factors could impact this including our use of ensembles of 4 ns trajectories (compared to single 16 ns runs) and Gaussian/RESP charges (as opposed to AM1-BCC). Overall though, it is important to retain at least four of the conserved water molecules in the binding site for the ESMACS calculations in order to obtain consistently good rankings across datasets. Moreover, the impact of adding water molecules differs between runs initiated with different starting structures, as shown in Table 7.

**Table 7:** Performance of 1traj and 2traj-ar MMPBSA based ESMACS protocols in reproducing experimental binding free energies incorporating different numbers of explicit water molecules.

Protocol	No. Water	2OSS				4BJX			
		cMUE*		r_s_		cMUE*		r_s_	
1traj	0	3.25	(0.51)	0.46	(0.18)	3.32	(0.53)	0.46	(0.18)
	1	3.59	(0.45)	0.52	(0.16)	3.62	(0.46)	0.46	(0.17)
	2	3.76	(0.47)	0.54	(0.16)	3.78	(0.48)	0.48	(0.17)
	3	3.91	(0.48)	0.51	(0.16)	3.88	(0.49)	0.48	(0.16)
	4	4.01	(0.50)	0.52	(0.16)	3.96	(0.50)	0.48	(0.16)
	5	4.11	(0.52)	0.52	(0.15)	4.03	(0.50)	0.47	(0.17)
2traj-ar	0	3.57	(0.54)	0.66	(0.11)	3.97	(0.44)	0.60	(0.13)
	1	3.70	(0.43)	0.65	(0.11)	4.14	(0.41)	0.64	(0.12)
	2	3.96	(0.45)	0.70	(0.11)	4.62	(0.47)	0.67	(0.12)
	3	4.28	(0.49)	0.68	(0.12)	5.04	(0.52)	0.67	(0.12)
	4	4.57	(0.51)	0.68	(0.12)	5.35	(0.56)	0.66	(0.12)
	5	4.79	(0.54)	0.68	(0.12)	5.59	(0.58)	0.66	(0.12)

Performance is measured by mean unsigned error (MUE) and Spearman’s rank coefficient (*r*_*s*_) (bootstrapped error provided in brackets). Results are provided for the combined diverse (DIV) and THQ datasets bound to protein models based on both PDBs 2OSS and 4BJX.

*MUE corrected for mean signed error in kcal mol^−1^.

The combined DIV + THQ 4BJX 1traj ranking shows only a consistent result, with no improvement, as the first 5 water molecules were incorporated, whereas in 2OSS the ranking improves from an *r*_*s*_ of 0.46 [CI: 0.16–0.84] to 0.54 [CI: 0.16–0.84] after the first two water molecules are included. In 1traj-ar the 4BJX results improve from a lower baseline rapidly whilst those from 2OSS remain consistent until 5 water molecules are added, at which point the results from both structures give an *r*_*s*_ of around 0.6. A similar pattern is seen in 2traj-ar, but with the peak performance at 2 water molecules of 0.70 [CI: 0.54–0.97]/0.67 [CI: 0.49–0.96] (2OSS/4BJX) as shown in Table 7. The increase in MUE which accompanies the improvement in correlation indicates that the effects are not uniform across all ligands. Marginal gains in correlation coefficient should not be over emphasized (as can be seen in Table 7, improvements are often within error); we rather wish to draw attention to the trend that inclusion of water molecules likely to be involved in mediating stable ligand-protein interactions improves (or at least does not degrade) calculation performance. The most important observation is that the addition of explicit water molecules improves the reproducibility of the ranking when using different starting models.

### Variational Entropy

**Figure 8:**
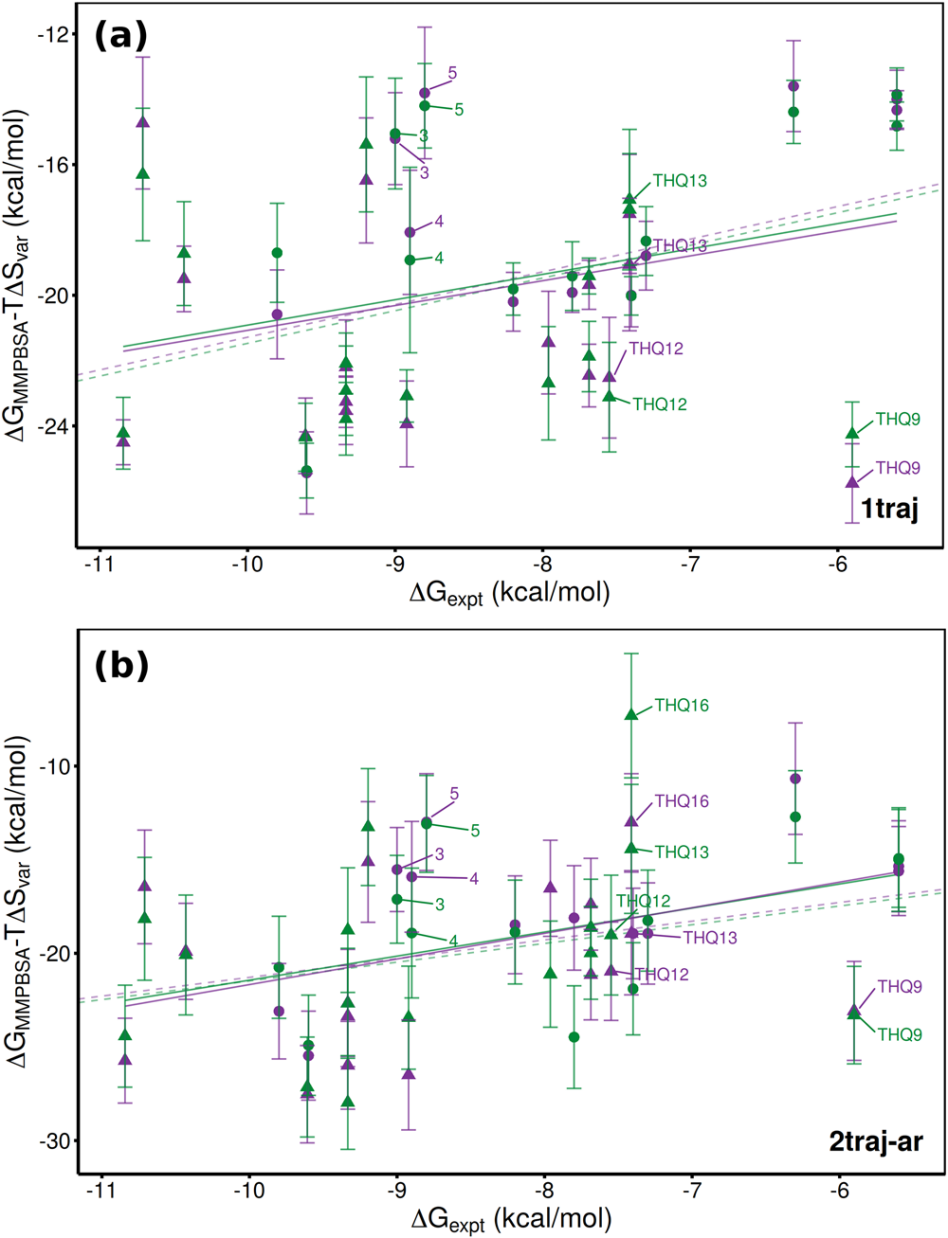
Comparison of experimental and computed binding affinities incorporating variational entropy for the combined DIV (circle) and THQ (triangle) dataset. Computational results obtained using (**a**) 1traj and (**b**) 2traj-ar MMPBSA based ESMACS protocols. Results are shown for simulations initiated from models based on PDBs 2OSS (green) and 4BJX (purple). Solid lines represent lines of best fit, dashed ones optimal correlations.

Accounting correctly, and computationally efficiently, for the entropic component of binding free energies remains a challenge for MMPBSA based computations. Here we investigated the use of the variational entropy technique on the ranking of different ESMACS protocols. In all cases the variational entropy was computed using the fluctuations from the 1traj simulations. As shown in Table 8 the inclusion of this term results in a reduction in the performance of all protocols in simulations based on both initial models. Furthermore, the incorporation of explicit water molecules into the receptor reduces this to an even greater extent. Looking in more detail we see that some compounds suffer a deterioration in prediction whilst others manifest an improvement. For instance, Fig. 8 shows that the three DIV outliers 3, 4 and 5 are closer to the trend line than in Fig. 5, whereas THQ12 and THQ13 are more poorly predicted. The entropic term is based on the variation in interaction energy during the complex simulation. As it compares versus the average it captures properties of the interaction energy surface. For molecules such as 6, 8, 9 and 10 that have few degrees of freedom, the interaction energy surface is likely to be steep, with small changes in conformation or translations leading to a rapid loss of interaction energy. Meanwhile, larger more flexible compounds such as 4 and 5 (which has a flexible benzhydryl core) can adapt to conformational changes of the receptor and maintain a favourable interaction energy, leading to a flatter potential surface. The results suggest that this entropic term is suited to the latter but not the former examples. Correctly capturing entropic contributions is key to obtaining truly reliable rankings in diverse datasets and further work in this area is required. Also, components of the MMPBSA calculation (particularly the surface area term) incorporate some entropic contributions and such double counting may account at least in part for the poor performance of variational entropy here.

**Table 8:** Performance of 1traj and 2traj-ar MMPBSA based ESMACS protocols in reproducing experimental binding free energies incorporating both variational entropy and different numbers of explicit water molecules.

Protocol	No. Water	2OSS				4BJX			
		cMUE*		r_s_		cMUE*		r_s_	
1traj	0	2.86	(0.34)	0.34	(0.20)	2.87	(0.41)	0.35	(0.20)
	2	4.66	(1.34)	0.19	(0.22)	4.37	(1.14)	0.24	(0.22)
	5	5.07	(1.35)	0.14	(0.21)	4.80	(1.39)	0.18	(0.21)
2traj-ar	0	3.35	(0.51)	0.43	(0.16)	3.39	(0.41)	0.46	(0.16)
	2	5.57	(1.37)	0.30	(0.19)	4.70	(0.99)	0.40	(0.19)
	5	5.57	(1.48)	0.35	(0.20)	5.35	(1.11)	0.46	(0.18)

Performance is measured by mean unsigned error (MUE) and Spearman’s rank coefficient (*r*_*s*_) (bootstrapped error provided in brackets). Results are provided for the combined diverse (DIV) and THQ datasets bound to protein models based on both PDBs 2OSS and 4BJX.

*MUE corrected for mean signed error in kcal mol^−1^.

## Discussion

In summary, we have investigated the influence of different analysis choices on the results of ensemble MMPBSA based free energy calculations. The basis of our tests are two datasets which cover common computational chemistry challenges - one which is based on a set of related ligands and the other a highly diverse set of ligands with differing binding modes. In order to obtain successful rankings across the two datasets we found it necessary to incorporate receptor and ligand strains. Using the 2traj-ar ESMACS protocol we obtained Spearman correlations of between 0.60 [CI: 0.46–0.91] and 0.66 [CI: 0.50–0.94] for two different starting structures despite differences in charge and scaffold in the ligands. The lower confidence bounds of both these estimates are comparable to the average correlation coefficient from the 1traj protocol 0.46 [CI: 0.16–0.84], suggesting the result is statistically significant despite the relatively modest size of the dataset (which contains a total of 27 ligands). It should be noted that increase in computational cost is minimal here as the only additional simulations required are of the ligand (which are much smaller than either complex or receptor) with the receptor energy replaced by a constant. Hence, for prospective day to day applications, we recommend accounting for both ligand and receptor strain through independent ligand simulations and either further simulation of the apo receptor (as in the 3 traj ESMACS protocol) or the use of an average value for the receptor energies (2traj-ar).

A key consideration in the use of binding free energy calculations in real world (industrial or clinical) settings is the reproducibility of the results. Other considerations include computational cost and calculation stability. ESMACS protocols offer advantages in both these regards as they make use of relatively simple and fast classical MD simulations compared to many parallel simulations of intermediate states as required in alchemical calculations of absolute binding free energies^44^. We have shown that the results obtained in this study are robust to changing the ligand charge generation protocol (to use AM1-BCC instead of Gaussian/RESP) and the forcefield used to parameterize the protein (from Amber ff14SB to ff99SBildn). The use of ensemble simulations is the key to obtaining this reproducibility as individual replicas in ensembles varied by as much as 15 kcal mol^−1^ (which is in line with our own and other groups previous results^9,16,18,45^). Despite this, performance differences were found for all initial protocols when simulations were initiated from different crystal structures.

This observation, along with the fact that some ligands which have very similar experimental binding energies were widely separated even using protocols which accounted for receptor flexibility (1traj-ar and 2traj-ar), prompted us to investigate potential enhancements of the pure MMPBSA protocol. Specifically, we looked at the inclusion of an explicit ligand hydration shell in the receptor and variational entropy which had previously been investigated for single replica simulations by Aldeghi *et al*.^22^ (though they also only investigated what we would term “1traj” calculations). These additional components capture chemical and physical features of the system neglected by MMPBSA but at minimal computational cost, a key consideration for practical binding affinity calculation applications. The entropy term reduced extreme outliers but at the expense of decreased overall ranking performance. This observation replicates that obtained by Aldeghi *et al*.^22^ for the DIV dataset bound to BRD4, although they found the term improved results for sensitivity based datasets including multiple proteins. When less than five water molecules were incorporated into the receptor our rankings were improved with the best ranking across the full dataset obtained using this in combination with the 2traj-ar protocol. The most important observation of our work, however, is that the inclusion of these bound water molecules considerably reduced the performance difference between simulations initiated from models based on different crystal structures. A criticism of continuum based methods is that they are incapable of capturing the effect of crucial water molecules, possible activity cliffs, etc, that are now a well understood feature of structure-activity relationship (SAR) landscapes and medicinal chemistry lead optimization. Here it is shown again how this challenge can be met, with the simple inclusion of explicit water molecules. Future work should address how to consider this in prospective application scenarios and in a wider range of protein targets.

The reason for the improved performance observed for the diverse datasets in this study is presumably due to the capture of interactions between the ligand and the closest of the four conserved water molecules also found in the binding site. This observation is in line with other work in which system dependent numbers of water molecules were found to improve rankings^46–49^ and the broader phenomenon of the impact of crucial water molecules on SAR landscapes. Incorporation of the water molecules was highly effective in differentiating the ligands with diverse binding modes but less effective in the set of related THQ-scaffold based compounds. The fact that our observations fit a general pattern, and that the level of explicit water hydration which improves results is similar to the number of conserved water molecules suggests that the approach can be applied more generally.

Overall we have shown that, for a diverse set of ligands, in order to deliver reproducible results from ESMACS (MMPBSA) calculations it is necessary to account for receptor and ligand strain and account explicitly for water molecules bound alongside ligands. Essential to obtaining these results is the use of ensemble simulations to generate meaningfully quantified uncertainties.

## Supplementary information

LaTeX Supplementary File

LaTeX Supplementary File

LaTeX Supplementary File

LaTeX Supplementary File

LaTeX Supplementary File

LaTeX Supplementary File

LaTeX Supplementary File

LaTeX Supplementary File

LaTeX Supplementary File

LaTeX Supplementary File

LaTeX Supplementary File

LaTeX Supplementary File

LaTeX Supplementary File

LaTeX Supplementary File

## Data Availability

Simulation input topologies and coordinates (alongside ligand parameters) for all protein-ligand systems and collated MMPBSA results are made available via Zenodo, 10.5281/zenodo.1484050. Trajectories are available from the corresponding author on reasonable request.

## References

[1] Paul, S. M. *et al*. How to improve R&D productivity: the pharmaceutical industry’s grand challenge. *Nature Reviews Drug Discovery* **9**, 203–214, https://www.nature.com/articles/nrd3078 (2010). 10.1038/nrd3078 20168317

[2] Mobley DL, Klimovich PV (2012). Perspective: Alchemical free energy calculations for drug discovery. The Journal of Chemical Physics.

[3] Mey Antonia S. J. S., Jiménez Jordi Juárez, Michel Julien (2017). Impact of domain knowledge on blinded predictions of binding energies by alchemical free energy calculations. Journal of Computer-Aided Molecular Design.

[4] Yin J (2017). Overview of the sampl5 host–guest challenge: Are we doing better?. J. Comput.-Aided Mol. Des..

[5] Ganesan, A., Coote, M. L. & Barakat, K. Molecular dynamics-driven drug discovery: leaping forward with confidence. *Drug Discovery Today* **22**, 249–269, http://www.sciencedirect.com/science/article/pii/S1359644616304147 (2017). 10.1016/j.drudis.2016.11.001 27890821

[6] Pérez-Benito, L., Keränen, H., van Vlijmen, H. & Tresadern, G. Predicting binding free energies of pde2 inhibitors. the difficulties of protein conformation. *Sci*. *Rep*. **8**, 10.1038/s41598-018-23039-5 (2018). 10.1038/s41598-018-23039-5 PMC5861043 29559702

[7] Keränen H (2017). Acylguanidine beta secretase 1 inhibitors: A combined experimental and free energy perturbation study. J. Chem. Theory Comput..

[8] Wan S (2017). Evaluation and characterization of trk kinase inhibitors for the treatment of pain: Reliable binding affinity predictions from theory and computation. Journal of Chemical Information and Modeling.

[9] Wan, S. *et al*. Rapid and reliable binding affinity prediction of bromodomain inhibitors: a computational study. *J*. *Chem*. *Theory Comput*. (2016). 10.1021/acs.jctc.6b00794 PMC5312866 28005370

[10] Wang L (2015). Accurate and Reliable Prediction of Relative Ligand Binding Potency in Prospective Drug Discovery by Way of a Modern Free-Energy Calculation Protocol and Force Field. Journal of the American Chemical Society.

[11] Sherborne B (2016). Collaborating to improve the use of free-energy and other quantitative methods in drug discovery. J. Comput.-Aided Mol. Des..

[12] Baker M (2016). 1,500 scientists lift the lid on reproducibility. Nature.

[13] Ioannidis JPAWhyMost (2005). Published Research Findings Are False. PLoS Med..

[14] Kollman PA (2000). Calculating structures and free energies of complex molecules: combining molecular mechanics and continuum models. Acc. Chem. Res..

[15] Aldeghi M, Heifetz A, BodkinJ MJ, Knapp S, Biggin PC (2016). Accurate calculation of the absolute free energy of binding for drug molecules. Chem. Sci..

[16] Wright DW, Hall BA, Kenway OA, Jha S, Coveney PV (2014). Computing clinically relevant binding free energies of HIV-1 protease inhibitors. J. Chem. Theory Comput..

[17] Wan S, Knapp B, Wright DW, Deane CM, Coveney PV (2015). Rapid, precise, and reproducible prediction of peptide–MHC binding affinities from molecular dynamics that correlate well with experiment. J. Chem. Theory Comput..

[18] Sadiq SK, Wright DW, Kenway OA, Coveney PV (2010). Accurate ensemble molecular dynamics binding free energy ranking of multidrug-resistant HIV-1 proteases. J. Chem. Inf. Model..

[19] Aldeghi, M., Heifetz, A., Bodkin, M. J., Knapp, S. & Biggin, P. C. Predictions of Ligand Selectivity from Absolute Binding free Energy Calculations. *J*. *Am*. *Chem*. *Soc*. **139**, 946–957, https://www.ncbi.nlm.nih.gov/pmc/articles/PMC5253712/ (2017). 10.1021/jacs.6b11467 PMC5253712 28009512

[20] Mobley DL, Gilson MK (2017). Predicting Binding Free Energies: Frontiers and Benchmarks. Annu. Rev. Biophys..

[21] Mobley, D. L. & Slochower, D. Mobleylab/Benchmarksets: Version 1.2, https://zenodo.org/record/839047 (2017).

[22] Aldeghi Matteo, Bodkin Michael J., Knapp Stefan, Biggin Philip C. (2017). Statistical Analysis on the Performance of Molecular Mechanics Poisson–Boltzmann Surface Area versus Absolute Binding Free Energy Calculations: Bromodomains as a Case Study. Journal of Chemical Information and Modeling.

[23] Sadiq SK (2008). Automated Molecular Simulation Based Binding Affinity Calculator for Ligand-Bound HIV-1 Proteases. J. Chem. Inf. Model..

[24] Balasubramanian, V., Treikalis, A., Weidner, O. & Jha, S. Ensemble Toolkit: Scalable and Flexible Execution of Ensembles of Tasks. *arXiv*:*1602*.*00678 [cs]*, http://arxiv.org/abs/1602.00678, ArXiv: 1602.00678 (2016).

[25] Merzky, A., Turilli, M., Maldonado, M., Santcroos, M. & Jha, S. Using Pilot Systems to Execute Many Task Workloads on Supercomputers. *arXiv*:*1512*.*08194 [cs]*, http://arxiv.org/abs/1512.08194, ArXiv: 1512.08194 (2015).

[26] Dakka, J. *et al*. High-throughput Binding Affinity Calculations at Extreme Scales. *arXiv*:*1712*.*09168 [cs]*, http://arxiv.org/abs/1712.09168, ArXiv: 1712.09168 (2017). 10.1186/s12859-018-2506-6 PMC6302294 30577753

[27] Wright DW, Coveney PV (2011). Resolution of Discordant HIV-1 Protease Resistance Rankings Using Molecular Dynamics Simulations. J. Chem. Inf. Model..

[28] Hall BA, Wright DW, Jha S, Coveney PV (2012). Quantized water access to the HIV-1 protease active site as a proposed mechanism for cooperative mutations in drug affinity. Biochemistry (Mosc.).

[29] Wan S, Coveney PV (2011). Rapid and accurate ranking of binding affinities of epidermal growth factor receptor sequences with selected lung cancer drugs. J. R. Soc. Interface.

[30] Hou T, Wang J, Li Y, Wang W (2011). Assessing the performance of the MM/PBSA and MM/GBSA methods. 1. The accuracy of binding free energy calculations based on molecular dynamics simulations. J. Chem. Inf. Model..

[31] Miller BR (2012). MMPBSA. py: an efficient program for end-state free energy calculations. J. Chem. Theory Comput..

[32] Case DA (2014). Amber 14.

[33] Genheden S, Kuhn O, Mikulskis P, Hoffmann D, Ryde U (2012). The normal-mode entropy in the MM/GBSA method: effect of system truncation, buffer region, and dielectric constant. J. Chem. Inf. Model..

[34] Wang, C., Greene, D., Xiao, L., Qi, R. & Luo, R. Recent Developments and Applications of the MMPBSA Method. *Frontiers in Molecular Biosciences* **4**, 10.3389/fmolb.2017.00087/full (2018). 10.3389/fmolb.2017.00087 PMC5768160 29367919

[35] Duan Lili, Liu Xiao, Zhang John Z.H. (2016). Interaction Entropy: A New Paradigm for Highly Efficient and Reliable Computation of Protein–Ligand Binding Free Energy. Journal of the American Chemical Society.

[36] Phillips JC (2005). Scalable molecular dynamics with NAMD. J. Comput. Chem..

[37] Case DA (2005). The Amber biomolecular simulation programs. J. Comput. Chem..

[38] Case D (2017). Amber 17.

[39] Maier JA (2015). ff14SB: improving the accuracy of protein side chain and backbone parameters from ff99SB. J. Chem. Theory Comput..

[40] Hornak V (2006). Comparison of multiple Amber force fields and development of improved protein backbone parameters. Proteins: Struct., Funct., Bioinf..

[41] Wang J, Wolf RM, Caldwell JW, Kollman PA, Case DA (2004). Development and testing of a general Amber force field. J. Comput. Chem..

[42] Frisch, M. J. *et al*. Gaussian 98 (Gaussian, Inc., 1998).

[43] Jakalian A, Jack DB, Bayly CI (2002). Fast, efficient generation of high-quality atomic charges. am1-bcc model: Ii. parameterization and validation. J. Comput. Chem..

[44] Bhati AP, Wan S, Hu Y, Sherborne B, Coveney PV (2018). Uncertainty Quantification in Alchemical Free Energy Methods. J. Chem. Theory Comput..

[45] Genheden S, Ryde U (2011). A comparison of different initialization protocols to obtain statistically independent molecular dynamics simulations. J. Comput. Chem..

[46] Zhu Yong-Liang, Beroza Paul, Artis Dean R. (2014). Including Explicit Water Molecules as Part of the Protein Structure in MM/PBSA Calculations. Journal of Chemical Information and Modeling.

[47] Maffucci Irene, Contini Alessandro (2013). Explicit Ligand Hydration Shells Improve the Correlation between MM-PB/GBSA Binding Energies and Experimental Activities. Journal of Chemical Theory and Computation.

[48] Genheden Samuel, Mikulskis Paulius, Hu LiHong, Kongsted Jacob, Söderhjelm Pär, Ryde Ulf (2011). Accurate Predictions of Nonpolar Solvation Free Energies Require Explicit Consideration of Binding-Site Hydration. Journal of the American Chemical Society.

[49] Wong Sergio, Amaro Rommie E., McCammon J. Andrew (2009). MM-PBSA Captures Key Role of Intercalating Water Molecules at a Protein−Protein Interface. Journal of Chemical Theory and Computation.

