# Supplementary figures and images for: Application of ESMACS binding free energy protocols to diverse datasets: Bromodomain-containing protein 4
Source: Sci Rep. 2019 Apr 12;9:6017. doi: 10.1038/s41598-019-41758-1 (PMC6461631; doi:10.1038/s41598-019-41758-1)

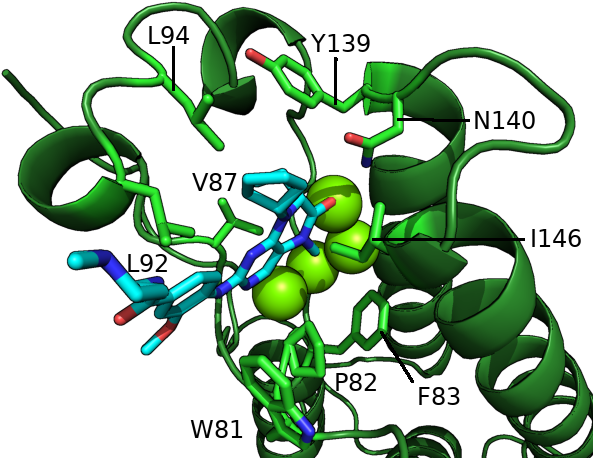

Supplement: Supplementary file 1 — LaTeX Supplementary File [file 41598_2019_41758_MOESM1_ESM.png]

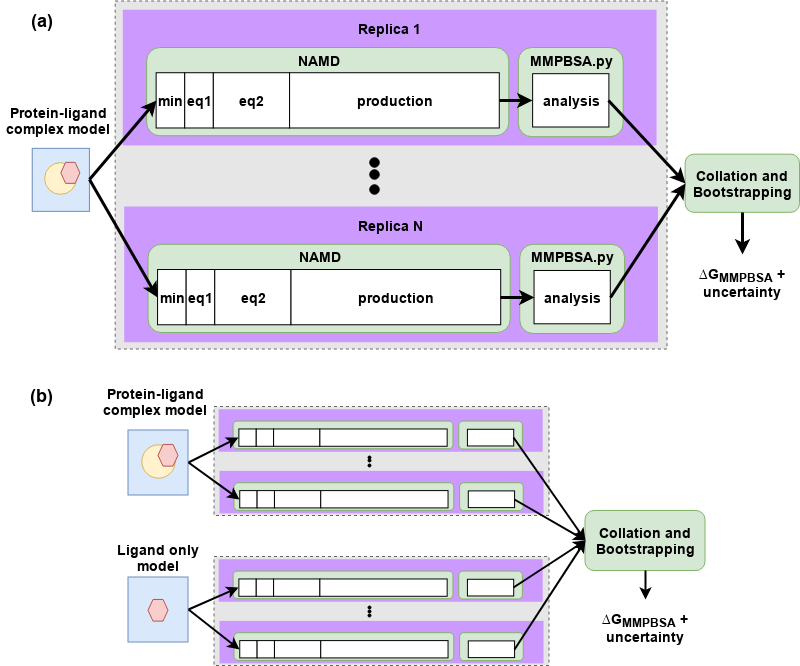

Supplement: Supplementary file 2 — LaTeX Supplementary File [file 41598_2019_41758_MOESM2_ESM.png]

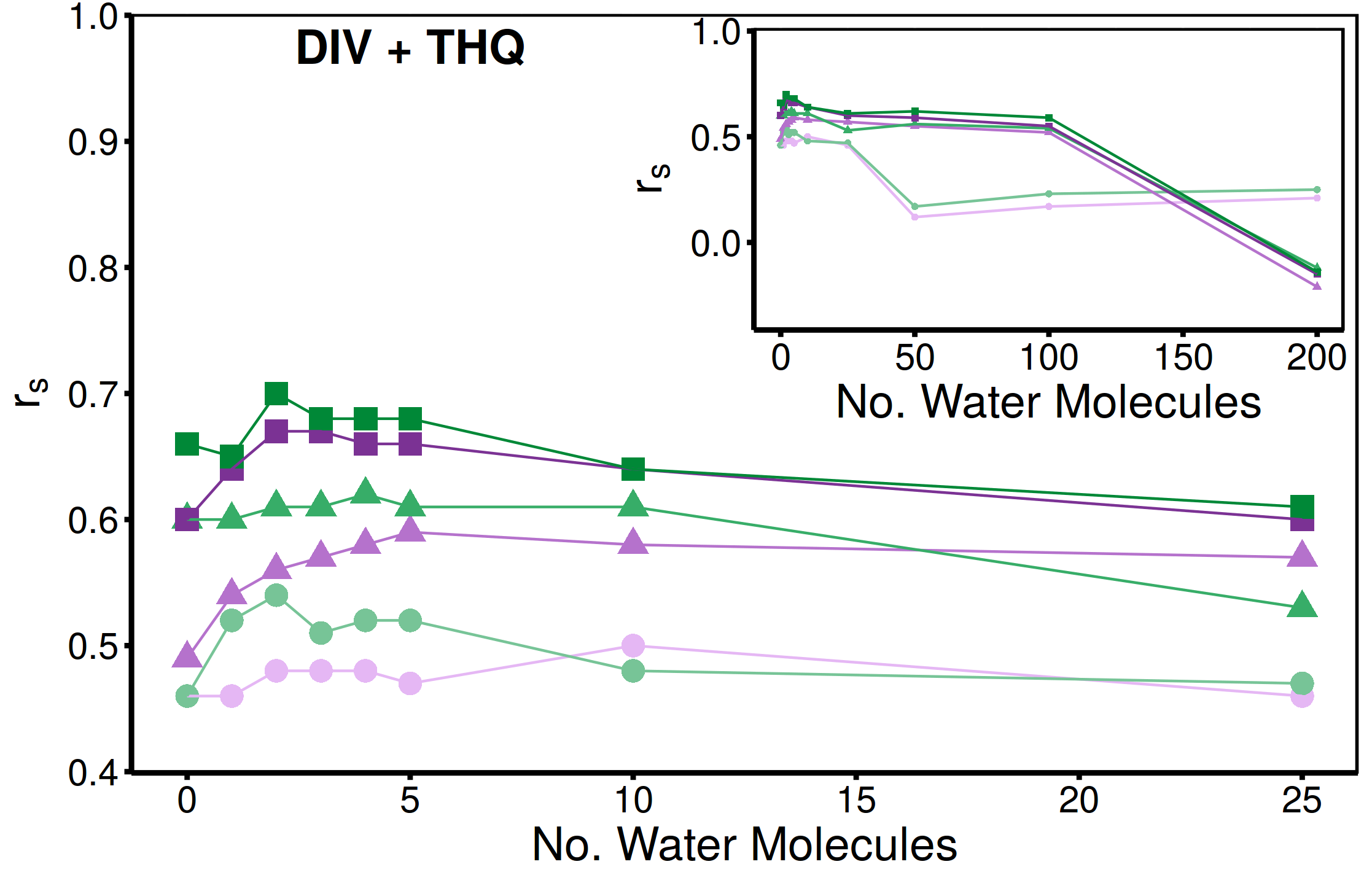

Supplement: Supplementary file 3 — LaTeX Supplementary File [file 41598_2019_41758_MOESM3_ESM.png]

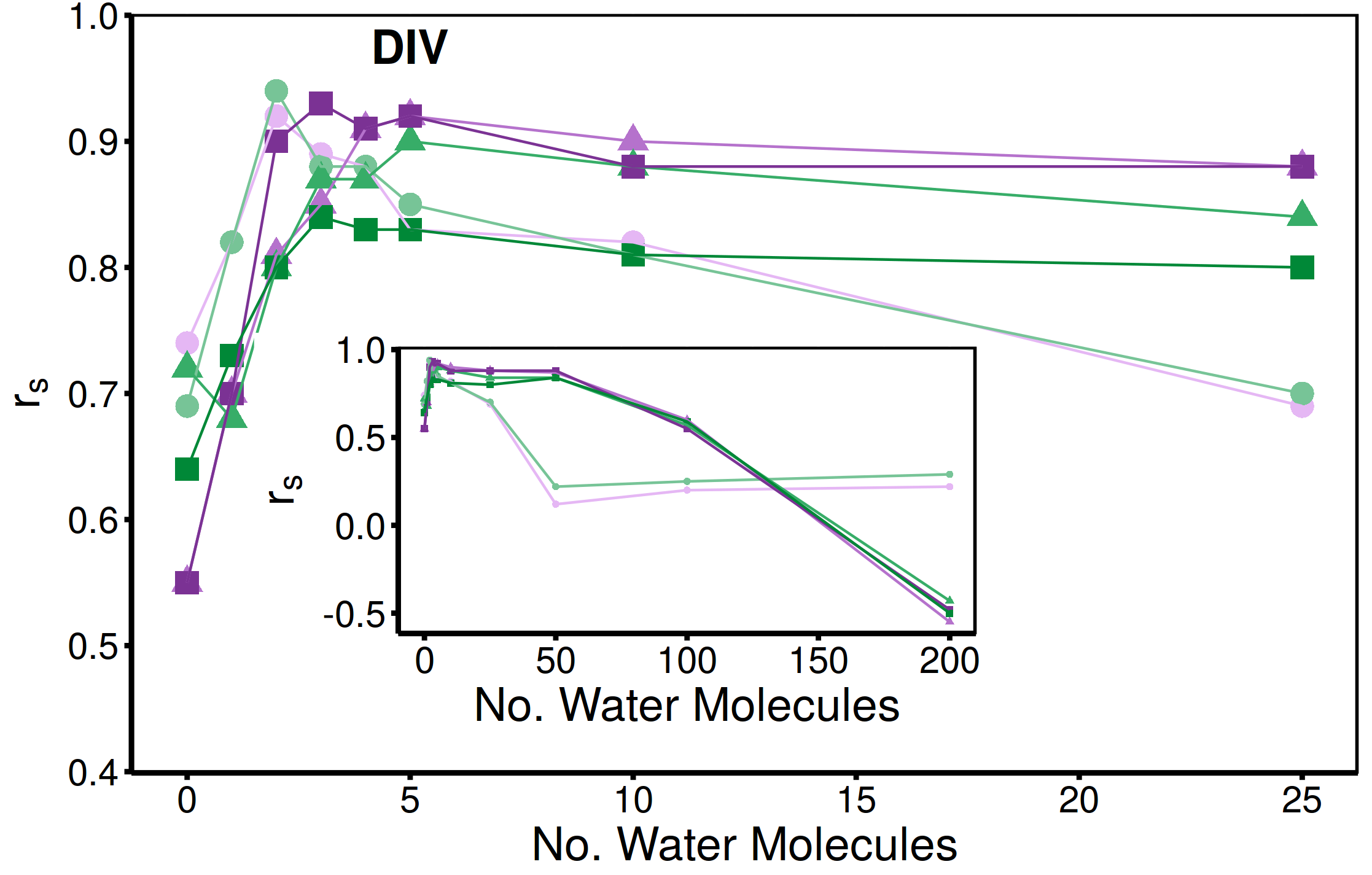

Supplement: Supplementary file 4 — LaTeX Supplementary File [file 41598_2019_41758_MOESM4_ESM.png]

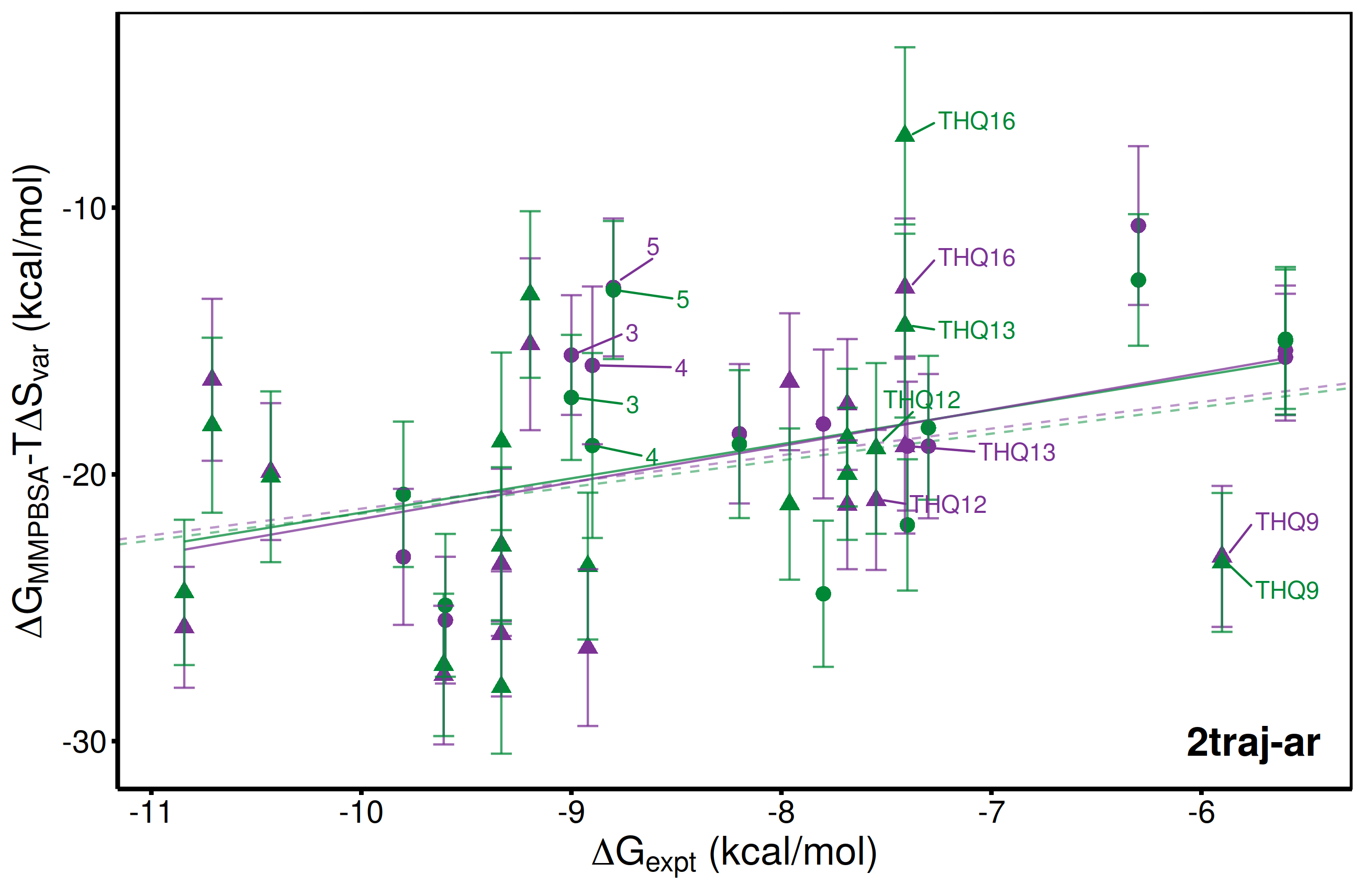

Supplement: Supplementary file 5 — LaTeX Supplementary File [file 41598_2019_41758_MOESM5_ESM.png]

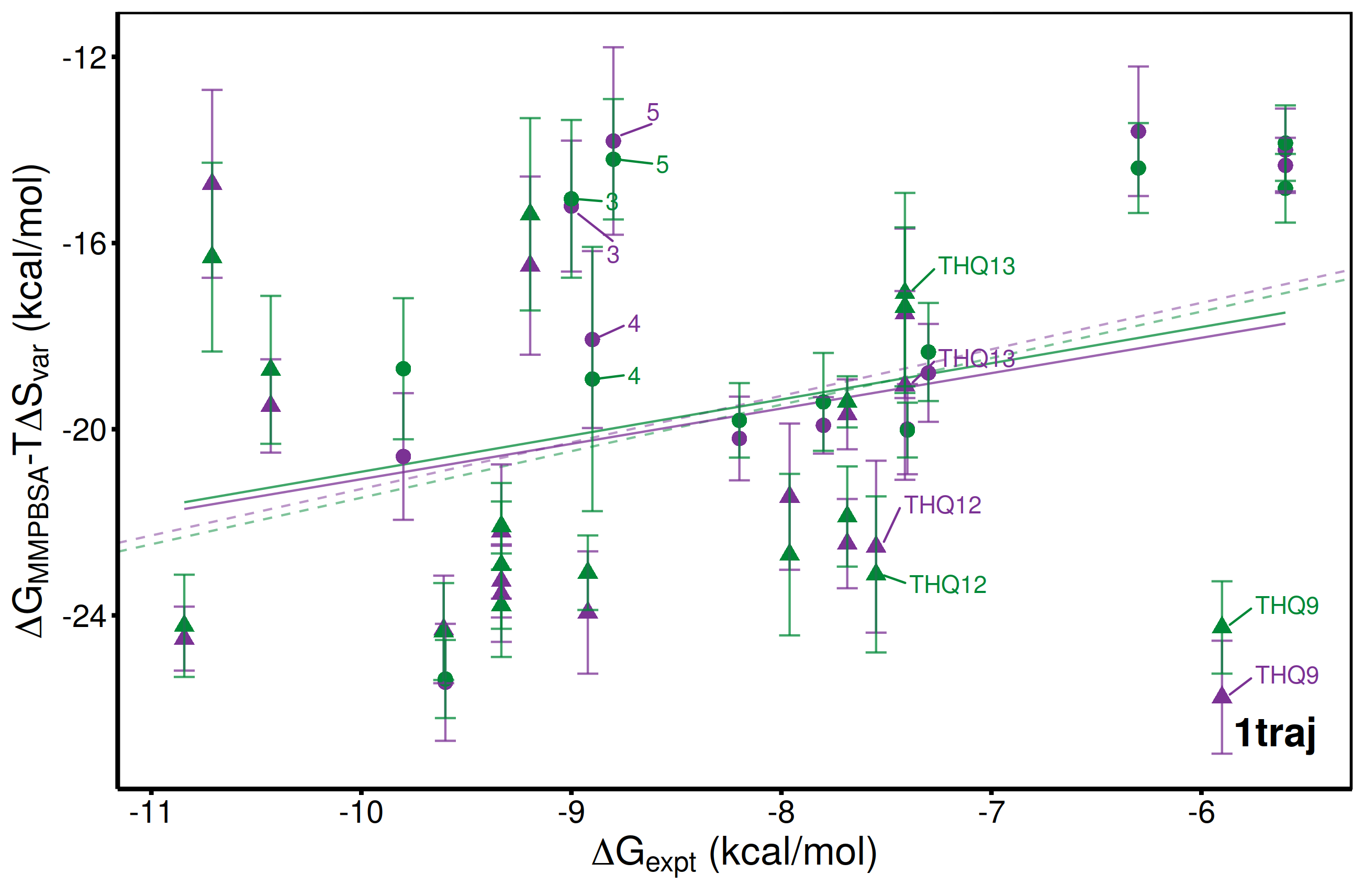

Supplement: Supplementary file 6 — LaTeX Supplementary File [file 41598_2019_41758_MOESM6_ESM.png]

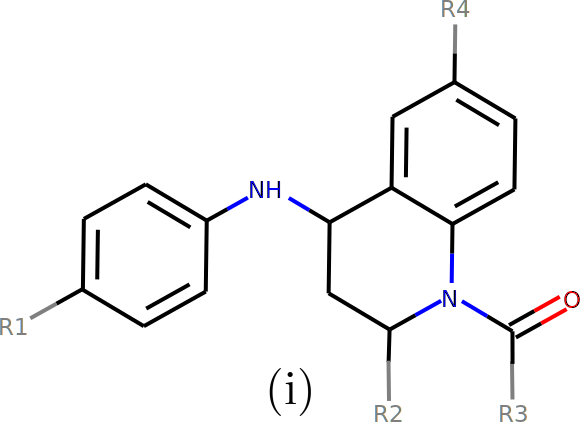

Supplement: Supplementary file 7 — LaTeX Supplementary File [file 41598_2019_41758_MOESM7_ESM.png]

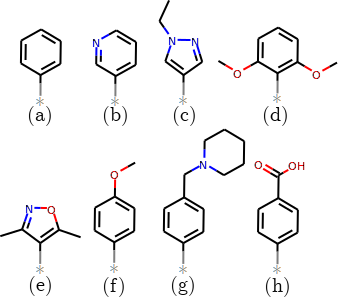

Supplement: Supplementary file 8 — LaTeX Supplementary File [file 41598_2019_41758_MOESM8_ESM.png]

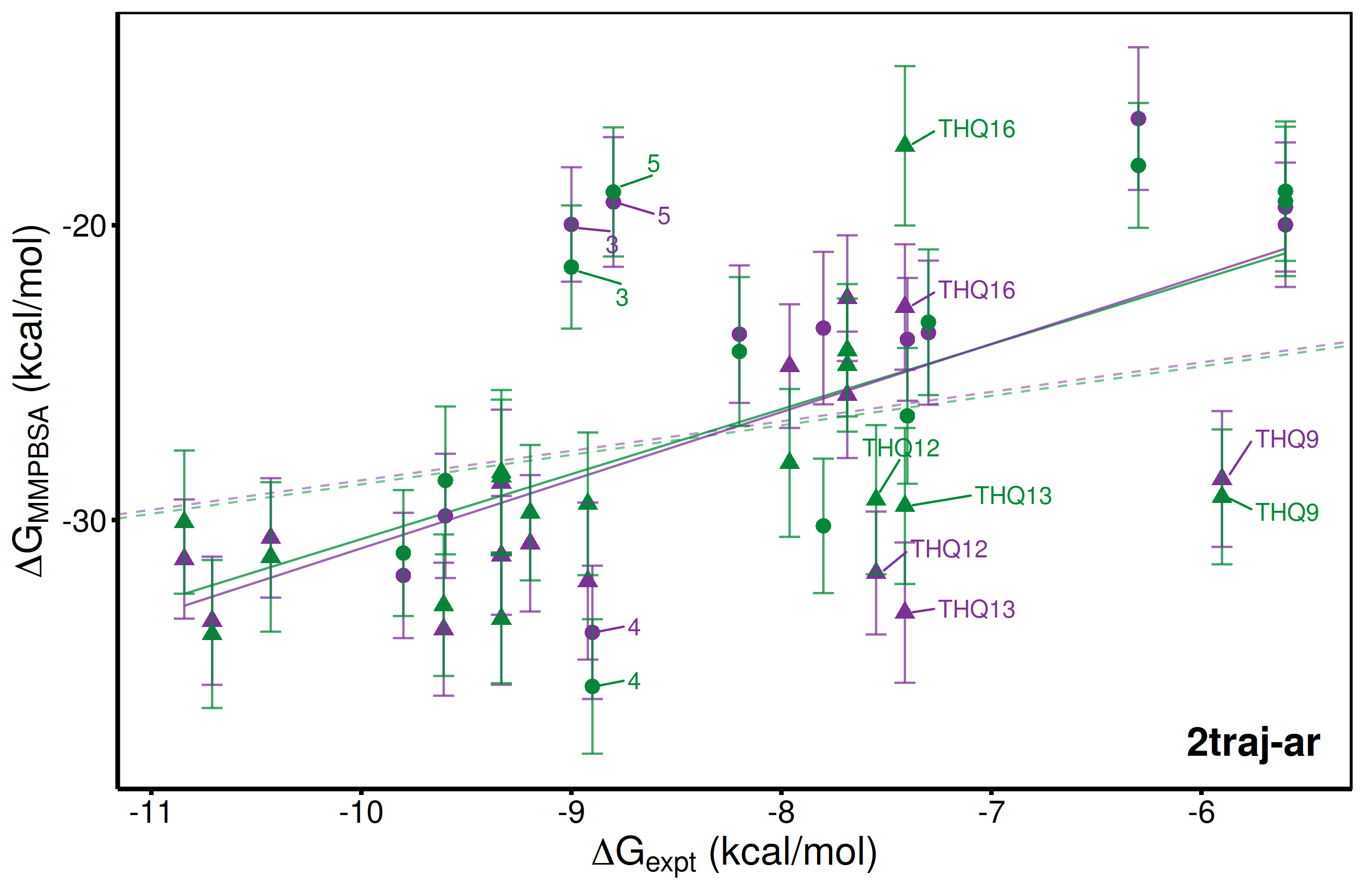

Supplement: Supplementary file 9 — LaTeX Supplementary File [file 41598_2019_41758_MOESM9_ESM.png]

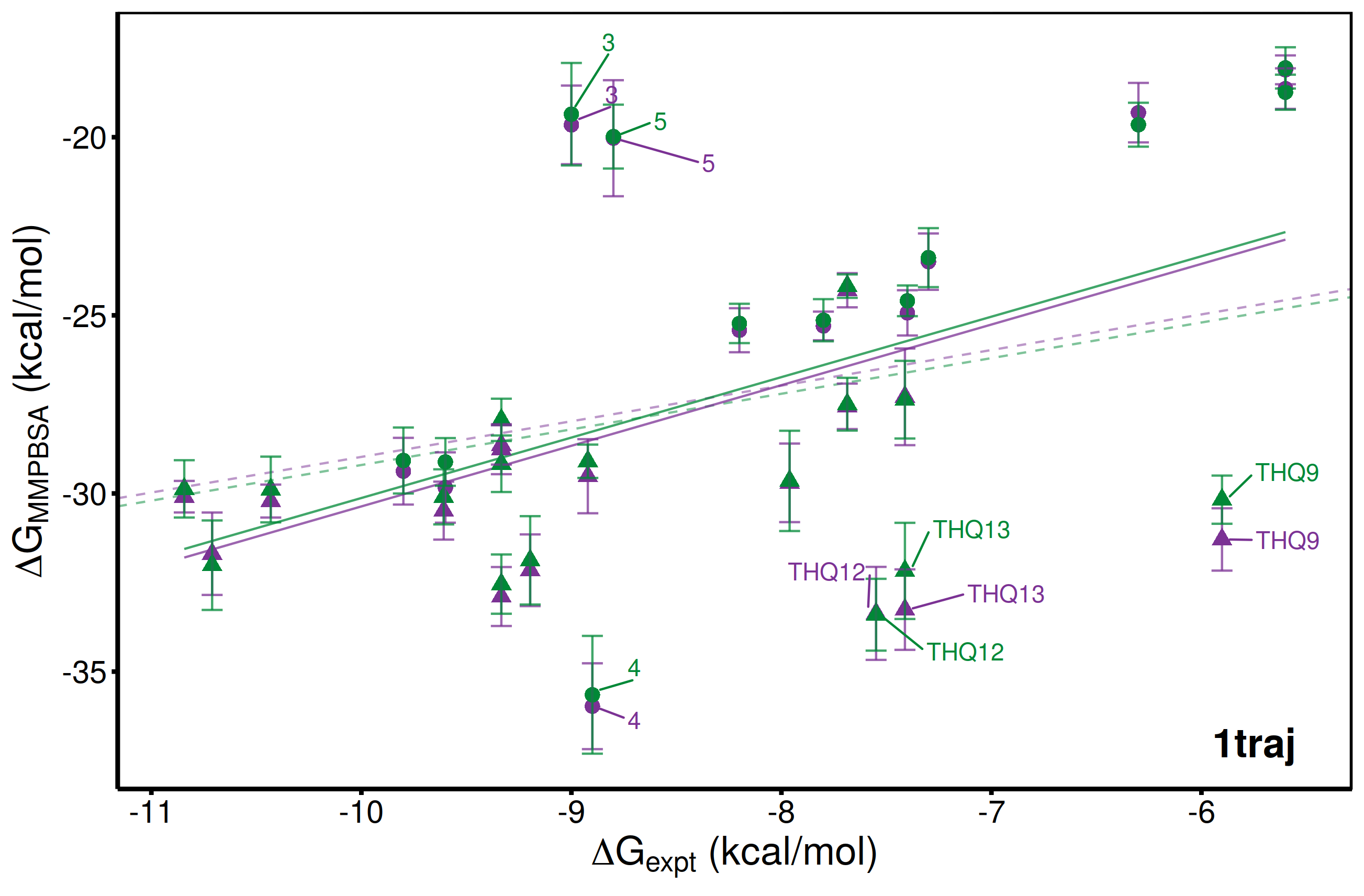

Supplement: Supplementary file 10 — LaTeX Supplementary File [file 41598_2019_41758_MOESM10_ESM.png]

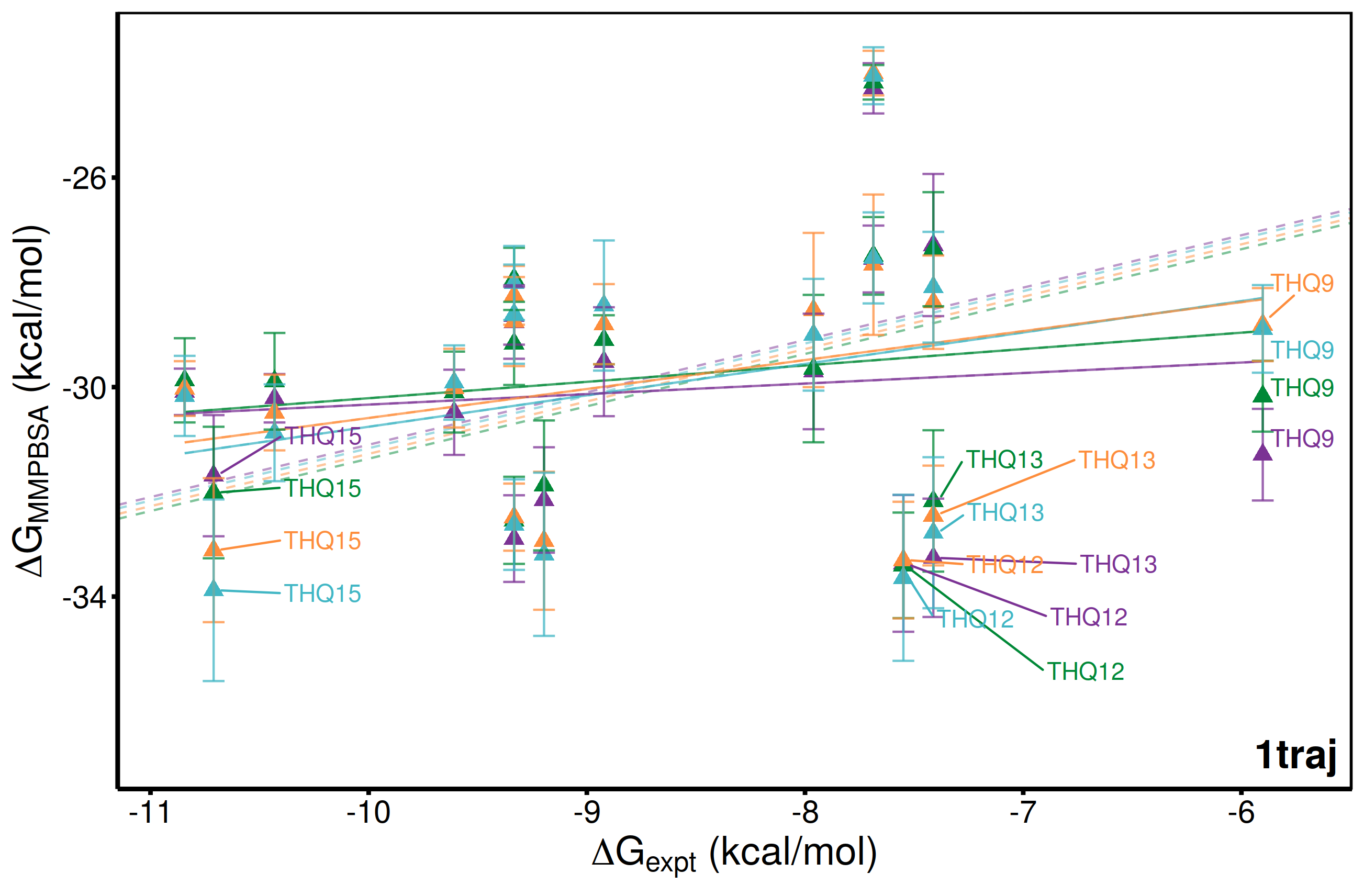

Supplement: Supplementary file 11 — LaTeX Supplementary File [file 41598_2019_41758_MOESM11_ESM.png]

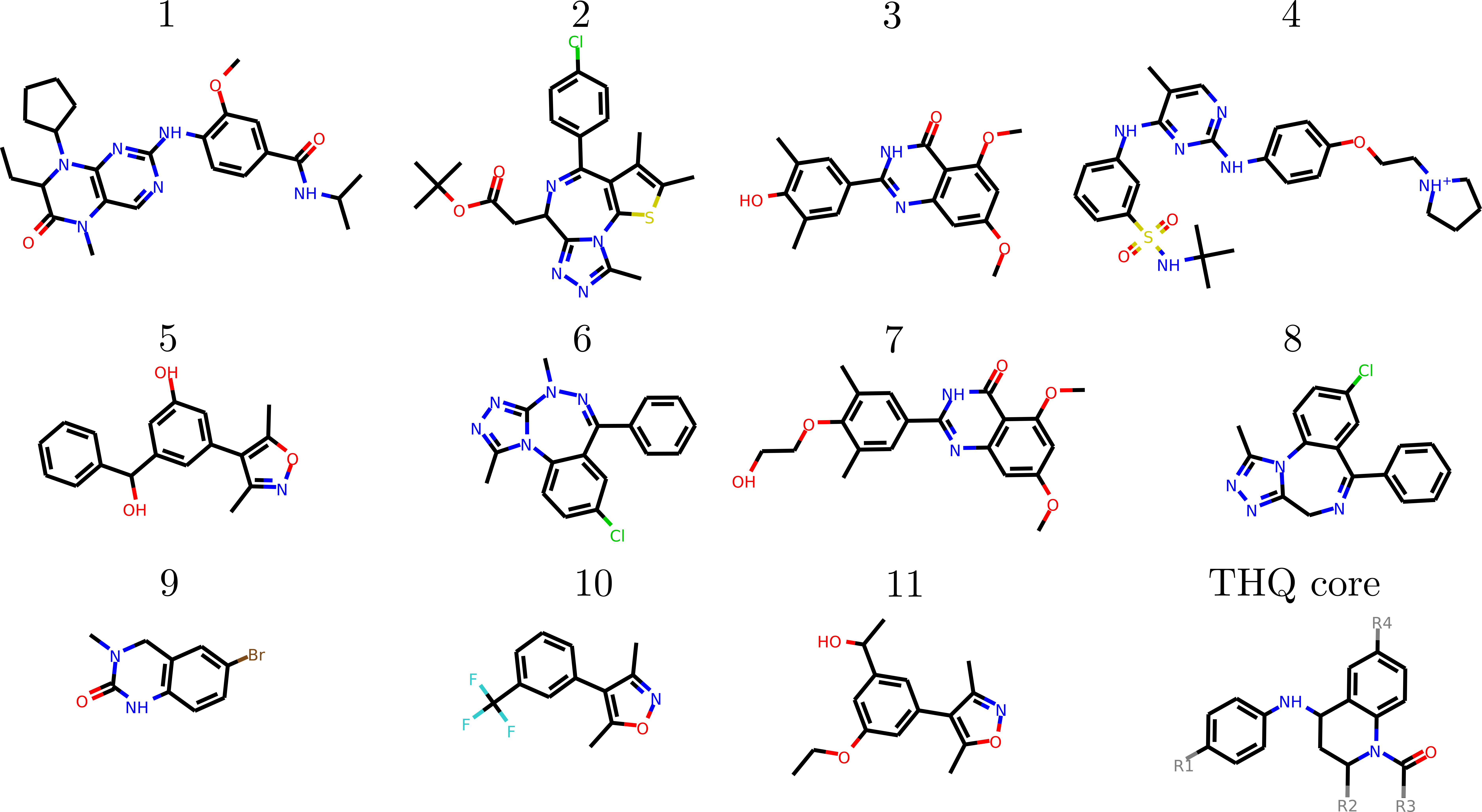

Supplement: Supplementary file 12 — LaTeX Supplementary File [file 41598_2019_41758_MOESM12_ESM.png]

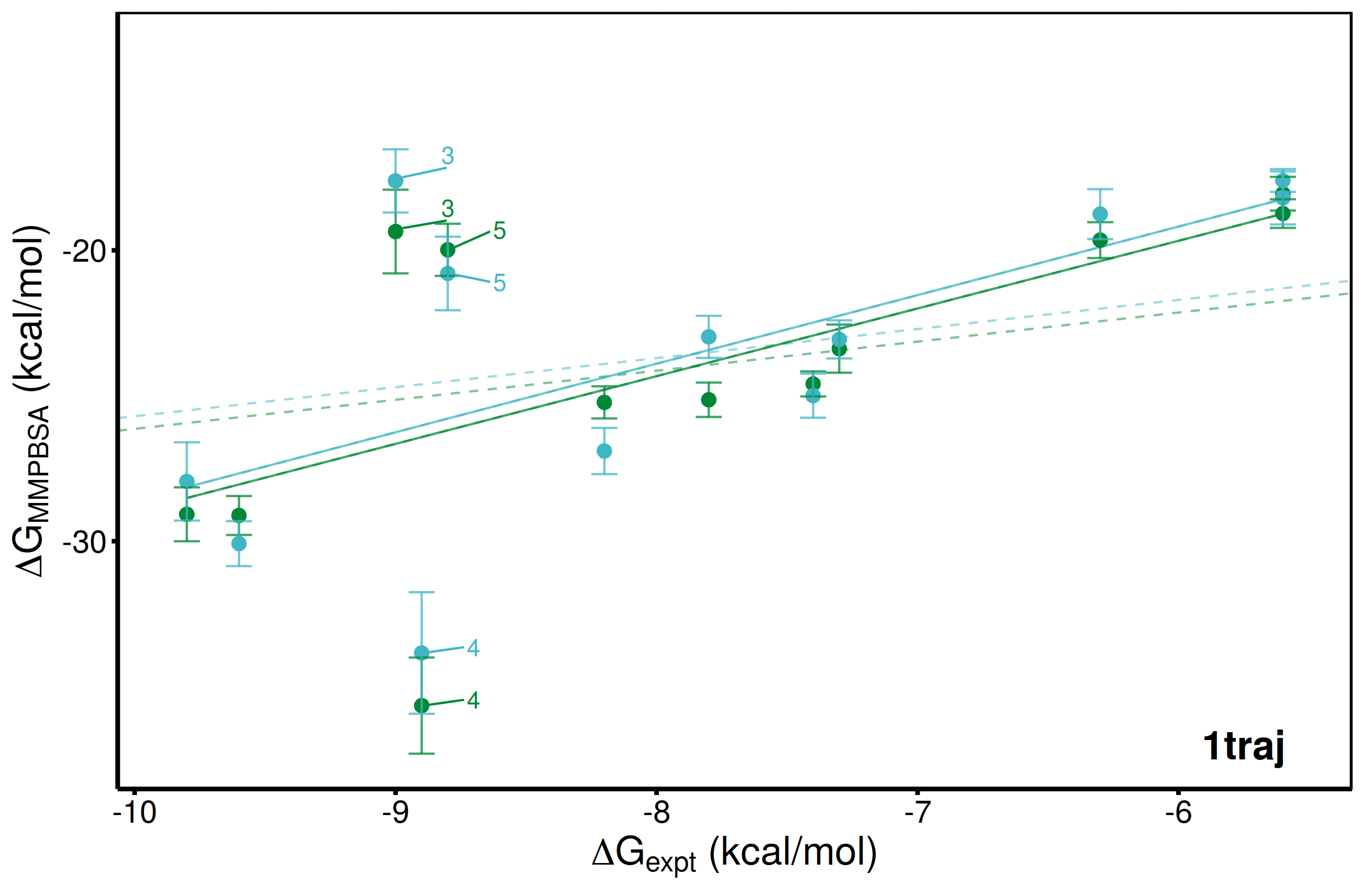

Supplement: Supplementary file 13 — LaTeX Supplementary File [file 41598_2019_41758_MOESM13_ESM.png]

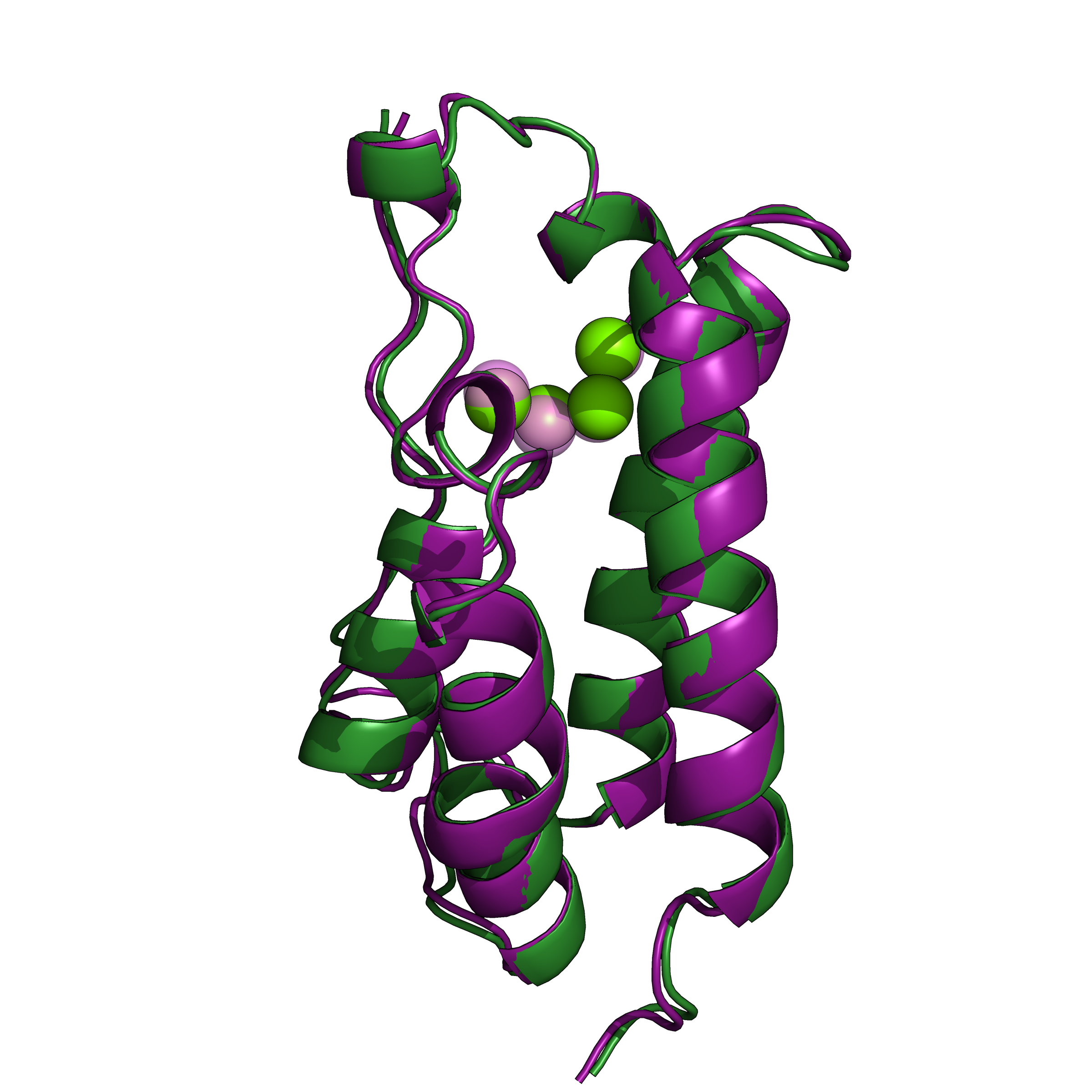

Supplement: Supplementary file 14 — LaTeX Supplementary File [file 41598_2019_41758_MOESM14_ESM.png]
